# Nonlinear Dependence in the Discovery of Differentially Expressed Genes

**DOI:** 10.5402/2012/564715

**Published:** 2012-04-12

**Authors:** J. R. Deller, Hayder Radha, J. Justin McCormick, Huiyan Wang

**Affiliations:** ^1^Department of Electrical and Computer Engineering, Michigan State University, 2120 EB, East Lansing, MI 48824, USA; ^2^Carcinogenesis Laboratory, Department of Molecular Biology and Biochemistry, Michigan State University, 341 FST, East Lansing, MI 48824, USA; ^3^College of Computer Science and Information Engineering, Zhejiang Gongshang University, 18 Xuezheng Street, Zhejiang Province Hangzhou, 310018, China

## Abstract

Microarray data are used to determine which genes are active in response to a changing cell environment. Genes are “discovered” when they are significantly differentially expressed in the microarray data collected under the differing conditions. In one prevalent approach, all genes are assumed to satisfy a null hypothesis, *ℍ*_0_, of no difference in expression. A *false discovery* (type 1 error) occurs when *ℍ*_0_ is incorrectly rejected. The quality of a detection algorithm is assessed by estimating its *number of false discoveries*, *𝔉*. Work involving the second-moment modeling of the *z*-value histogram (representing gene expression differentials) has shown significantly deleterious effects of intergene expression correlation on the estimate of *𝔉*. This paper suggests that nonlinear dependencies could likewise be important. With an applied emphasis, this paper extends the “moment framework” by including third-moment skewness corrections in an estimator of *𝔉*. This estimator combines observed correlation (corrected for sampling fluctuations) with the information from easily identifiable null cases. Nonlinear-dependence modeling reduces the estimation error relative to that of linear estimation. Third-moment calculations involve empirical densities of 3 × 3 covariance matrices estimated using very few samples. The principle of entropy maximization is employed to connect estimated moments to *𝔉* inference. Model results are tested with BRCA and HIV data sets and with carefully constructed simulations.

## 1. Introduction

 This work is motivated by analytical challenges that arise in the use of microarray data to discover genes that are differentially expressed across experimental conditions such as control and treatment. Although the discussion centers around this genomics task, the developed methods are quite general and should be useful in other multiple-testing applications in which there is substantial dependence among test measures, and in which a small sample size may cause significant fluctuations in statistics employed in the testing. The specific aim of this work is to develop a reliable estimator of the *number of false discoveries* (type I errors—denoted *𝔉*) in a multiple-testing problem in such an adverse setting.

The classic and contemporary literature in cell biology, and the more recent literature in genomics (both in print and posted on the Internet), is replete with tutorial information at all levels about cell anatomy and physiology, and genomics, as well as the microarray technology deployed in the present application. A good entry point for accessing information about contemporary developments in the genomics field is the web site of the US National Genome Research Institute [1]. The papers by Page et al. [2] and Wang [3] provide relatively current reviews of microarray technology and methods. A brief description of the biological aspects of the genomics application underlying this work is found in Appendix A of this paper.

In the gene-discovery application, each gene is tested against a null hypothesis, *ℍ*_0_, that the gene is *not* differentially expressed across experimental conditions. All genes are initially assumed to satisfy *ℍ*_0_ in this analysis, and *𝔉* is estimated conservatively. This “all-null” presumption is consistent with this application in which *ℍ*_0_ is true for a vast majority of the genes in any experiment. Beyond the gene detection problem, however, this presumption is realistic in many practical applications of large-scale testing in which the prior probability of null cases, say *π*_0_, is large, and in which the goal is to identify a small set of interesting “nonnull” cases [4]. With *π*_0_ ≈ 1, it is also possible to impose *identifiability* (the strongly justified assumption that a given gene satisfies *ℍ*_0_) on some of the genes, yielding crucial information with which to condition the estimation of *𝔉* [5].

One of the earliest reports of research using the microarray (or “gene chip”) appeared in a paper by Schena et al. in *Science* in 1995 [6]. Generally speaking, research that employs the microarray to analyze gene expression data has one (or both) of the following underlying aims: the discovery of gene *coexpression*, or the discovery of gene *differential expression*. To the extent that these problems have been investigated separately, the coexpression problem has frequently been addressed by clustering methods (e.g., [7–13]), whereas differential expression has been studied using variations of classical statistical hypothesis testing (e.g., [5, 14]). Whereas differential-expression/hypothesis-testing research was, and is, concerned with expression in response to differing cell conditions (normal versus pathology, medical treatment versus control, etc.), the early coexpression/clustering research was often focused on phenotypic manifestations of the gene expression.

As the technology has matured, the dichotomy suggested above has blurred with many current applications of the microarray involving “hybrid” research questions into both differential and coexpression. Application areas include discovery and exploration of gene regulatory systems, tissue and tumor classification, biomarker prediction, discovery and reverse engineering of gene expression networks—not to mention the microarray's deployment in the study of protein synthesis, metabolism, evolution, and other areas related to cell biology. Technical approaches to these problems have gone well beyond classical clustering and hypothesis-testing methods. Indeed, in the past few years, *statistical* (i.e., “nonclustering”) methods to address the coexpression problem have been reported (e.g., [15, 16]), while the hybrid of the two problems—that of detecting and analyzing *differentially coexpressed* genes—has been researched using an ever-increasing number of methods including clustering with complex and dynamic feature selection methods, image transformation and processing of expression data, biclustering, graph and network theory, hypothesis testing, and other statistical approaches (e.g., [17–28]). In this paper, we return to the focused problem of detecting differential expression across treatment conditions, but it will become clear—as it has to the community working on this problem—that differential expression cannot be studied independently of coexpression.

Early work on classical statistical techniques for microarray-based gene discovery is summarized in the 2002 review paper by Pan [29]. Initially, it was customary to treat gene expression outcomes as realizations of independent random variables (RVs). More recent papers, however—notably, those of Owen [30], Efron [31], and Pawitan et al. [32]—caution researchers of the detrimental effects of correlated gene-expressions on the validity of “discovered” genes. In particular, it was reported that highly correlated tests increase the variance of *𝔉* (or, its normalized counterpart, the *false discovery rate*, $\dot{𝔉}≝𝔉/G_{*}$, where *G*_∗_ is the number of “discovered” genes), thus making estimates of *𝔉* less reliable. In particular, high variance renders the average, ${\hat{\mu }}_{𝔉}\approx \mu_{𝔉}=ℰ\{𝔉\}$, an unreliable estimator of *𝔉* [30]. Among many causes, intergene correlation is attributable to coexpressed genes [4] and to unmodeled factors that introduce systematic effects across genes [33, 34]. As a result, for most real data, the assumption of independence or weak dependence among gene expressions is unfounded, and methods treating correlation are necessary [35, 36].

Accordingly, there has been significant recent interest in improving statistical gene detection methods in light of this detrimental correlation. For example, Storey et al. [37] present an approach to the notion of sharing information across *t* scores, which they describe as “borrowing strength across the tests” for a potential increase in statistical power. Tibshirani and Wasserman [38] discuss a quantity called the “correlation-shared” *t*-statistic and derives theoretical bounds on its performance. Hu et al. [39] examine the covariance structure of the expression data and discover benefits of linking coexpression and differential expression in a distance measure—thus, moving toward the “hybrid” problem described above.

Recent research into the hybrid differential coexpression problem has also yielded results and methods that could ultimately benefit the differential expression problem. Because the differential coexpression research is often concerned with differing phenotypes, rather than with different treatment conditions, two given research efforts involving differential coexpression might seek answers to different sets of genetic questions through expression data. Like the “dual conditions researchers,” however, the “phenotype” researchers have encountered their own forms of confounding dependencies, notably the relative gene locations, the expression time sequencing, and phase information (e.g., [40–42]). Papers have been published addressing these issues, including the exposition of new statistical approaches—for example, “CorScor” developed by Dettling et al. [21], the “ECF-statistic” of Lai et al. [22], “the gene-set coexpression analysis” of Choi and Kendziorski [15]—as well as new clustering methods—for example, the web-based expression analyzer of Xiang et al. [43], high-order preclustering method of Wong et al. [44], and the “BioSym” distance measure of Bandyopadhyay and Bhattacharyya [45]. A recent review of clustering methods in genomics appears in the paper by Dalton et al. [46]. A more general examination of the performance of classifiers of microarray expressions appears in the paper by Ancona et al. [47].

The present paper returns to the problem of gene discovery by statistical hypothesis testing, but with the concern for the effects of nonlinear dependencies on (the estimation of) the number of false results. Empirical work below provides cogent evidence that accounting for intergene correlation alone does not sufficiently mitigate the adverse effects of dependency. Recent work by Hu et al. [48] has shown the importance of accounting for nonlinear dependence in imputing missing values in microarray data. Modeling nonlinear dependencies is a challenging problem, and the present work makes only a modest—nevertheless, empirically significant—step into the realm of nonlinear dependence by modeling the third-moment characteristics of the quantity *𝔉*. In principle, the proposed extension admits any order moment, but computational constraints limit the present developments. However, even the single step to a third-moment extension under severe sampling fluctuations is very challenging, and, in spite of this modest modeling enhancement, it is a hard-won extension yielding significantly improved estimates for a range of real and simulated examples (see Section 5).

Thus, a central finding of this work is that null statistic histogram approaches can be improved by including third-moment skewness corrections. Advancing the techniques to model higher order dependencies is challenging, but the effort could have a substantial payoff. Errors in gene detection are expensive in financial terms, but the derailing of biomedical research resulting from a false gene discovery could be profoundly costly in many ways. Even modest improvements in genomic techniques are potentially very significant.

## 2. Notation and Terminology

Because this paper has a practical aim, we will assume, without comment, “friendly” mathematical conditions such as existence of distributions, measurability, and sure convergence of integrals. Even so, the mathematical developments in this paper necessarily involve extensive notation and we strive for consistency and clarity in its use. Quantities are generally formulated as RVs unless stated otherwise. This excludes obviously deterministic quantities like sequence indices, integers defined in the abstract (e.g., the number of microarrays, “*M*”), and statistical expectations. Precise formulations require that probability distributions ordinarily be denoted formally as, for example, *p*_*v*_1_,*v*_2__(*ξ*_1_, *ξ*_2_) for the joint distribution of RVs *v*_1_ and *v*_2_, but the more common abusive notation “*p*(*v*_1_, *v*_2_)” is more expedient in a few cases. The notation *p*(·) may denote either a discrete or continuous (i.e., density) distribution, depending, of course, on the RV(s) being modeled. The meaning should be clear in context. We deliberately allow this ambiguity because it avoids some notational awkwardness as discrete distributions are fitted with densities. On the other hand, the notation *ℙ*(*A*) is used to denote a probability assignment to a measurable event *A*.

Many developments in this paper are centered on second-order statistical concepts. It is important to carefully define terminology used in this regard, since the vocabulary has nuanced differences across disciplines. The elementary notation for scalar RVs in Table 1 is standard and is used conventionally in this paper. A caveat arises in the discussion of related matrices, however. The term “correlation matrix” is used in this paper in a way consistent with its use in many statistical developments, but not in a way that is universal across disciplines. The following definitions are used throughout this paper.

**Definitions 1 .**

Consider a random vector **v** ^*T*^ = [*v*_1_ ⋯ *v*_*G*_] with mean vector ***μ***_**v**_≝*ℰ*{**v**}. Then, the *covariance matrix* associated with **v** is defined as

(1) $$\begin{array}{c} {\text{Σ}}_{v}≝E\left\{\left(v-\mu_{v}\right){\left(v-\mu_{v}\right)}^{T}\right\}\in R^{G\times G}, \end{array}$$

in which the (*i*, *j*) element is *φ*(*v*_*i*_, *v*_*j*_). The *correlation matrix *of **v** is

(2) $$\begin{array}{c} R_{v}≝E\left\{S^{-1}\left(v-\mu_{v}\right){\left(v-\mu_{v}\right)}^{T}S^{-1}\right\}, \end{array}$$

in which the (*i*, *j*) element is *φ*(*v*_*i*_, *v*_*j*_) and **S** is a diagonal matrix with (*i*, *i*) element = *σ*_*v*_*i*__, the standard deviation of the *i*th RV, *v*_*i*_.

In this paper, *the term “correlation matrix” will refer to the definition in* (2). On the contrary, in much of the engineering literature, the outer product *ℰ*{**v** **v** ^*T*^} = Σ_**v**_ + ***μ***_**v**_ ***μ***_**v**_^*T*^ is called the *(spatial) correlation matrix*. In this case the (*i*, *j*) element of the matrix is the scalar correlation *ℰ*{*v*_*i*_ *v*_*j*_}. In our definition the elements are correlation coefficients, which are, in fact, normalized covariances. One significant implication of this fact is that mean values of RVs have no effect on either matrix. This should be kept in mind in the developments to follow. 

## 3. Problem Formulation

*G* genes are to be studied using *M* microarray experiments. The expression values are recorded in an *G* × *M* matrix, **X** = [*x*_*gm*_]. For analytical purposes, the expression quantities *x*_*gm*_ are generally RVs. Each of the *M* microarray experiments takes place under one of two conditions (indexed by *k* = 1  or  2) such as control and treatment. These two subsets of the data are called *treatment groups* in the paper. There are *M*_*k*_ samples (i.e., microarrays) in treatment group *k*. Based on the evidence in **X**, we seek to identify a “small” number, *G*_∗_ ≪ *G*, of genes that are significantly differentially expressed across the two treatment groups. One widely used strategy (e.g., [5, 14]) is to posit that each of the genes, for *g* = 1,2,…*G* satisfies a *null hypothesis*,

(3) $$\begin{array}{cc} H_{0,g}\text{ : } & \;\text{Gene}\;g\;\text{is}\;not\;\text{differentially}\;\text{expressed}\\ & \;\text{in}\;\text{the}\;\text{two}\;\text{treatment}\;\text{groups}. \end{array}$$

All *G* genes are tested against this hypothesis using two-sample null statistics *z*_1_, *z*_2_,…, *z*_*G*_ [14]. The magnitudes of *z*_*g*_ scores establish a gene ranking, and the *G*_∗_ genes with the largest scores are reported as statistically significant discoveries.

Clearly, the list of *G*_∗_ discovered genes is only meaningful to the extent that *𝔉* is very small. Of course, *𝔉* can only be estimated since the state of any gene (i.e., whether or not it should be “discovered”) is unknown. Strong causal relationships among genes give rise to highly correlated *z*_*g*_ scores and greatly complicate the estimate of *𝔉* [30, 31]. Moreover, in spite of their declining cost, microarrays are still a relatively expensive technology. Consequently, the number of microarrays, *M*, in an experiment is usually smaller than number of genes, *G*, by as much as four orders of magnitude. Typically, existing microarrays record expression data for at least a few thousand genes. The fact that *M* ≪ *G* further complicates the problem because the knowledge about the underlying gene-gene correlation structure is critically sparse in the observations. At the same time, the consequences of correlation on differential analysis cannot be overlooked [35]. In fact, the present work will suggest that even nonlinear dependencies must be accounted for in order to properly estimate *𝔉*. Theoretical justifications for this contention are given momentarily.

This paper develops a moment-based estimator of *𝔉* by giving the null *z* histogram a stochastic interpretation. The observed null counts are viewed as realizations of a more fundamental random model shaped by inter-*z*_*g*_ dependence. A small zero-symmetric bin in the space of *z* statistics is designated as the *center area*, and it is posited that no *z*_*g*_ scores from “nonnull” genes fall in this range. Then, the null count in the center area, say *C*, is observable, and by conditioning *𝔉* on *C*, the variance of *𝔉* can be reduced significantly [5, 49]. We relate *𝔉* to *C* through the discrete joint distribution *p*(*𝔉*, *C*). To obtain an approximation of *p*(*𝔉*, *C*), we estimate its first three moments, then fit the maximum entropy function (Appendix B). This approach inherently yields an estimate of the conditional distribution *p*(*𝔉*∣*C*). A large number of estimates of the distribution of $\dot{𝔉}$ would theoretically be more useful than a point estimate because of the noisy nature of large-scale inferences [30]. Compared to histogram-curve-fitting techniques like empirical null [5], however, the present approach enjoys the attractive feature that covariance is separately estimated, and then explicitly incorporated into the inference.

Efron [31] reports that RVs *𝔉* and *C* are found to be extremely negatively correlated in a number of real experiments. He provides an explanation for this finding, then employs these insights to develop a Poisson-model-based second-order estimator of *𝔉* which, like the present approach, relies on the center area concept. While Efron's work is extremely important, his own research has gone on to show that purely second-order *𝔉* estimates suffer from over- and under-estimation effects. The second-moment estimates of *𝔉* reported later in the present paper (see Section 5), as well as those in the cited Efron paper, all show these adverse effects. There are three contributory factors: (i) *𝔉* is bounded below by zero, (ii) the mean of *𝔉* is small, and (iii) intergene covariance causes the variance of *𝔉* to inflate. All of these factors suggest that skewness corrections—reflecting nonlinear dependence—are vital.

## 4. Methods

### 4.1. Moments of the Joint Distribution *P*(*𝔉*, *C*)

#### 4.1.1. Count Models

The process begins by transforming test *t* statistics to *z* values as *z*_*g*_ = *P*_*𝒢*_*u*__^−1^{*P*_0_(*t*_*g*_)}, *g* = 1,…, *G*, where *P*_0_ is the putative null cumulative distribution function (c.d.f.) of the test statistic, and *P*_*𝒢*_*u*__^−1^ is the inverse c.d.f. of the unit normal density, *p*_*𝒢*_*u*__ ≡ *𝒢*(0,1). The *z* values, modeled as RVs, provide the analytical convenience of multivariate normal form while describing the joint *t*-statistic behavior. We formally define the fundamental quantities:

(4) $$\begin{array}{c} F≝\#\left\{z_{g}:\;z_{g}\leq \delta \cap H_{0,g}\;\;\text{is}\;\text{true}\right\},\\ C≝\#\left\{z_{g}:\;\left|z_{g}\right|\leq c\cap H_{0,g}\;\text{is}\;\text{true}\right\}, \end{array}$$

in which #{*𝒮*} denotes the number of elements in the discrete set {*S*}. *𝒵*⊆ℝ is the sample space of *z* values. The interval *𝒵*_*C*_≝{*z* ∈ *𝒵* : | *z*_*g*_ | < *c*} corresponding to count *C* is called the *center area*, and the semi-infinite interval *𝒵*_*𝔉*_≝{*z* ∈ *𝒵* : *z* ≤ *δ*} associated with count *𝔉* is the *left tail area*. For proper comparison with Efron's results [31], we work with a left tail area; however, the present approach can employ right- or double-sided tail areas equally well.

The premise that very few nonnull *z*_*g*_ scores fall in *𝒵*_*C*_ and, hence, that *C* is practically observable is of prime importance. A similar assumption plays a central role in the literature on estimating the proportion of null genes, as in, for example, papers by Efron [31], Pawitan et al. [32], and Langaas et al. [50]. The *empirical null* approach [5] relies on similar reasoning. We exploit the observability of *C* to: (i) estimate the moments of *p*(*𝔉*, *C*), (ii) use them to infer the distribution (estimate) $\hat{p}(𝔉,C)$, and then (iii) report (an estimated) *p*(*𝔉*∣*C*) which in turn could be used to find an estimator of *𝔉* conditioned upon *C*. Initially, all cases are treated as null. Improvement is possible by estimating *π*_0_ [50, 51].

#### 4.1.2. Assumptions

**Assumptions**

The following assumptions underlie these developments:

1. *π*_0_ is large, say *π*_0_ ≥ 0.9 (Efron discusses this bound in [4]).
2. *z*_*g*_ is a unit normal variate [~*𝒢*(0,1)] for all *g* = 1,2,…, *G*.
3. The *z* scores are jointly Gaussian to the third order (but *not* uncorrelated).
4. Recall that *x*_*gm*_ [element (*g*, *m*) of the expression matrix **X**] denotes (the RV model for) the expression of gene *g* on microarray *m*. Let *x*_*g*•_ denote the marginal RV for *x*_*gm*_, that is, the model for the expression outcomes of gene *g*. The realizations of *x*_*g*•_ are the elements of row *g* of an observed **X**. Then, it is assumed that *φ*(*z*_*g*_, *z*_*g*′_) = *ρ*(*z*_*g*_, *z*_*g*′_) ≈ *ρ*(*x*_*g*•_, *x*_*g*′•_) for all *g*, *g*′ (justified below).

#### 4.1.3. The *z*-Value Histogram

 It is convenient and computationally efficient to obtain the moments of *p*(*𝔉*, *C*) using the moments of the *z*-value histogram. We seek central moments because they facilitate working with the maximum entropy distribution (Appendix B). The moment estimation is carried out as follows. *𝒵* is partitioned into *B* disjoint bins, *𝒵* = ∪_*b*=1_^*B*^ *𝒵*_*b*_, where the *b*th bin has center *z* ^[*b*]^ and width Δ (constant with *b*). Then, the *z-histogram bin counts* are

(5) $$\begin{array}{c} Y_{b}=\#\left\{z_{m}:\;z_{g}\in Z_{b}\right\}=\overset{G}{\underset{g=1}{\sum }}I_{b}\left(z_{g}\right),\;\;\text{for}\;\;b=1,\ldots ,B, \end{array}$$

in which *I*_*b*_(*z*_*g*_) is the indicator function for the event “score *z*_*g*_ falls in bin *𝒵*_*b*_.”

Consider bin *b* with count *Y*_*b*_ for some *b* ∈ {1,…, *B*}. The *mean* of count *Y*_*b*_ is

(6) $$\begin{array}{cc} \mu \left(Y_{b}\right) & ≝E\left\{Y_{b}\right\}=E\left\{\overset{G}{\underset{g=1}{\sum }}I_{b}\left(z_{g}\right)\right\}\\ & =\overset{G}{\underset{g=1}{\sum }}P\left(z_{g}\in Z_{b}\right)=G\overset{z^{\left[b\right]}+(\Delta /2)}{\underset{z^{\left[b\right]}-(\Delta /2)}{\int }}p_{G_{u}}\left(\xi \right)\;d\xi \\ & \approx \hat{\mu }\left(Y_{b}\right)≝G\Delta p_{G_{u}}\left(z^{\left[b\right]}\right),\;\;\text{where}\;p_{G_{u}}\left(\xi \right)\equiv G\left(0,1\right). \end{array}$$

The *second-order joint central moment covariance *of the pair (*Y*_*b*_, *Y*_*b*′_), where *b* may equal *b*′, is

(7) $$\begin{array}{cc} \varphi \left(Y_{b},Y_{b\prime }\right) & ≝E\left\{\left[Y_{b}-\mu \left(Y_{b}\right)\right]\left[Y_{b^{\prime }}-\mu \left(Y_{b^{\prime }}\right)\right]\right\}\\ & =E\left\{\overset{G}{\underset{g=1}{\sum }}I_{b}\left(z_{g}\right)\overset{G}{\underset{g^{\prime }=1}{\sum }}I_{b^{\prime }}\left(z_{g^{\prime }}\right)\right\}-\mu \left(Y_{b}\right)\mu \left(Y_{b\prime }\right)\\ & =\underset{g\neq g^{\prime }}{\sum }P\left(z_{g}\in Z_{b},z_{g^{\prime }}\in Z_{b^{\prime }}\right)\\ & \;+\underset{g}{\sum }P\left(z_{g}\in Z_{b},z_{g}\in Z_{b^{\prime }}\right)-\mu \left(Y_{b}\right)\mu \left(Y_{b^{\prime }}\right). \end{array}$$

Because of the bivariate normality of *z*_*g*_ and *z*_*g*′_, (7) can be approximated by:

(8) $$\begin{array}{c} \hat{\hat{\varphi }}\left(Y_{b},Y_{b^{\prime }}\right)≝\overset{}{\underset{g\neq g^{\prime }}{\sum }}\Delta^{2}{p_{G}}_{\;}\left(z^{\left[b\right]},z^{\left[b^{\prime }\right]};0,{\text{Σ}}^{\left[g,g^{\prime }\right]}\right)\\ \;-\hat{\mu }\left(Y_{b}\right)\left[\hat{\mu }\left(Y_{b^{\prime }}\right)+\delta_{bb^{\prime }}\right], \end{array}$$

where *δ*_*bb*′_ is the Kronecker delta, and *p*_*𝒢*__  _(*ζ*_1_, *ζ*_2_; 0, Σ^[*g*,*g*′]^) ≡ *𝒢*(0, Σ^[*g*,*g*′]^) is the bivariate Gaussian density with mean vector 0 = [0 ⋯ 0]^*T*^, and covariance matrix (equivalent to the correlation matrix in this case)

(9) $$\begin{array}{cc} {\text{Σ}}_{z}^{\left[g,g^{\prime }\right]} & ≝E\left\{\left[\begin{array}{c} z_{g}\\ z_{g^{\prime }} \end{array}\right]\left[\begin{array}{cc} z_{g} & z_{g^{\prime }} \end{array}\right]\right\}\\ & =\left[\begin{array}{cc} 1 & \rho \left(z_{g},z_{g^{\prime }}\right)\\ \rho \left(z_{g^{\prime }},z_{g}\right) & 1 \end{array}\right]=R_{z}^{\left[g,g^{\prime }\right]}. \end{array}$$

That is, defining the vector of arguments **ζ**≝[*ζ*_1_ *ζ*_2_]^*T*^,

(10) $$\begin{array}{cc} & p_{G}\left(\zeta ;0,{\text{Σ}}_{z}^{\left[g,g^{\prime }\right]}\right)\\ & \;\;=\frac{1}{2\pi {\left|{\text{Σ}}_{z}^{\left[g,g^{\prime }\right]}\right|}^{1/2}}\;\\ & \;\;\;\times \exp\left\{-\frac{1}{2}\zeta^{T}{\left({\text{Σ}}_{z}^{\left[g,g^{\prime }\right]}\right)}^{-1}\zeta \right\}\\ & \;\;=\frac{1}{2\pi \sqrt{1-\rho^{2}\left(z_{g},z_{g^{\prime }}\right)}}\\ & \;\;\;\times \exp\left\{-\frac{\zeta_{1}^{2}-2\rho \left(z_{g},\;z_{g\prime }\right)\zeta_{1}\zeta_{2}+\zeta_{2}^{2}}{2\left[1-\rho^{2}\left(z_{g},z_{g^{\prime }}\right)\right]}\right\}, \end{array}$$

where |·| denotes the determinant. In the approximation (8), the density in (10) is evaluated at *ζ*_1_ = *z* ^[*b*]^ and *ζ*_2_ = *z* ^[*b*′]^.

As reflected in the second line of (10), the covariance matrix Σ_*z*_^[*g*,*g*′]^ is fully specified by a a single-scalar parameter, *ρ*(*z*_*g*_, *z*_*g*′_) for each *g*, *g*′ pair. Thus, we can express the Gaussian density in (10) as being parameterized by this autocorrelation coefficient for the given *z*-score pair, *p*__*𝒢*__[**ζ**; 0, *ρ*(*z*_*g*_, *z*_*g*′_)]. Now, suppose that we can derive an *empirical density* [52], say *q*_*ρ*_*z*__(·), over the interval [−1,1], fitted to the discrete set of $\left(\begin{array}{c} G\\ 2 \end{array}\right)$ autocorrelation coefficients, {*ρ*(*z*_*g*_, *z*_*g*′_), 1 ≤ *g*, *g*′ ≤ *G*}. This empirical distribution allows the summation over gene indices in (10) to be replaced by a continuous computation. This smoothed computation represents a further approximation of *φ*(*Y*_*b*_, *Y*_*b*′_), which is stated in the form of a lemma below. This result is similar to those of Owen [30, Theorem 1] and Efron [31, Lemma 2].

**Lemma 1 .**

Let *q*_*ρ*_*z*__(*ξ*) denote the empirical density of *ρ*_*z*_, derived from the $\left(\begin{array}{c} G\\ 2 \end{array}\right)\;$ *z*-pair sample correlation coefficients, *ρ*(*z*_*g*_, *z*_*g*′_), 1 ≤ *g*,  *g*′ ≤ *G*. Then, the second joint central moment of a histogram count pair (*Y*_*b*_, *Y*_*b*′_), where *b* may equal *b*′, is approximated by

(11) $$\begin{array}{cc} \varphi \left(Y_{b},Y_{b^{\prime }}\right)\approx \hat{\varphi }\left(Y_{b},Y_{b^{\prime }}\right) & ≝{\left[G!\right]}_{2}\Delta^{2}Q_{\rho_{z}}\left(z^{\left[b\right]},z^{\left[b^{\prime }\right]}\right)\\ & \;-\hat{\mu }\left(Y_{b}\right)\left[\hat{\mu }\left(Y_{b^{\prime }}\right)+\delta_{bb^{\prime }}\right], \end{array}$$

where [*G*!]_*ℓ*_≝*G*(*G* − 1)⋯(*G* − [*ℓ* − 1]),   for  1 ≤ *ℓ* ≤ *G*, and

(12) $$\begin{array}{cc} Q_{\rho_{z}}\left(\zeta_{1},\zeta_{2}\right) & ≝\overset{+1}{\underset{-1}{\int }}\frac{q_{\rho_{z}}\left(\xi \right)}{2\pi \sqrt{1-\xi^{2}}}\\ & \;\times \exp\left\{-\;\frac{\zeta_{1}^{2}-2\xi \zeta_{1}\zeta_{2}+\zeta_{2}^{2}}{2\left(1-\xi^{2}\right)}\right\}\text{d}\xi . \end{array}$$

Further, the *third joint central moment* of a triplet (*Y*_*b*_, *Y*_*b*′_, *Y*_*b*′′_), where two or more indices may be equal, is:

(13) $$\begin{array}{cc} & \gamma \left(Y_{b},Y_{b^{\prime }},Y_{b^{\prime \prime }}\right)\\ & \;≝E\{\left[Y_{b}-\mu \left(Y_{b}\right)\right]\\ & \;\;\;\;\times \left[Y_{b^{\prime }}-\mu \left(Y_{b^{\prime \prime }}\right)\right]\left[Y_{b^{\prime }}-\mu \left(Y_{b^{\prime \prime }}\right)\right]\}\\ & \;=E\left\{Y_{b}Y_{b^{\prime }}Y_{b^{\prime \prime }}\right\}-\mu \left(Y_{b}\right)\mu \left(Y_{b^{\prime }}\right)\mu \left(Y_{b^{\prime \prime }}\right)\\ & \;\;\;-[\mu \left(Y_{b}\right)\varphi \left(Y_{b^{\prime }},Y_{b^{\prime \prime }}\right)\\ & \;\;\;\;\;+\mu \left(Y_{b^{\prime }}\right)\varphi \left(Y_{b},Y_{b^{\prime \prime }}\right)\\ & \;\;\;\;\;+\mu \left(Y_{b^{\prime \prime }}\right)\varphi \left(Y_{b},Y_{b^{\prime }}\right)], \end{array}$$

where

(14) $$\begin{array}{cc} & E\left\{Y_{b}Y_{b\prime }Y_{b^{\prime \prime }}\right\}\\ & \;=E\left\{\overset{G}{\underset{g=1}{\sum }}I_{b}\left(z_{g}\right)\overset{G}{\underset{g^{\prime }=1}{\sum }}I_{b^{\prime }}\left(z_{g^{\prime }}\right)\overset{G}{\underset{g^{\prime \prime }=1}{\sum }}I_{b^{\prime \prime }}\left(z_{g^{\prime \prime }}\right)\right\}\\ & \;=\underset{g\neq g^{\prime }\neq g^{\prime \prime }}{\sum }P\left(z_{g}\in Z_{b},z_{g^{\prime }}\in Z_{b^{\prime }},z_{g^{\prime \prime }}\in Z_{b^{\prime \prime }}\right)\\ & \;\;+\delta_{bb^{\prime \prime }}\underset{g\neq g^{\prime \prime }}{\sum }P\left(z_{g}\in Z_{b},z_{g^{\prime \prime }}\in Z_{b^{\prime \prime }}\right)\\ & \;\;+\delta_{bb^{\prime }}\underset{g\neq g^{\prime }}{\sum }P\left(z_{g}\in Z_{b},z_{g^{\prime }}\in Z_{b^{\prime }}\right)\\ & \;\;+\delta_{b^{\prime }b^{\prime \prime }}\underset{g^{\prime }\neq g^{\prime \prime }}{\sum }P\left(z_{g^{\prime }}\in Z_{b^{\prime }},z_{g^{\prime \prime }}\in Z_{b^{\prime \prime }}\right)\\ & \;\;+\delta_{bb^{\prime }b^{\prime \prime }}\underset{g}{\sum }P\left(z_{g}\in Z_{b}\right), \end{array}$$

in which *δ*_*bb*′*b*′′_ is the Kronecker sequence over *ℤ* × *ℤ* × *ℤ*.

The assumed trivariate normality of the *z* scores implies that the joint distribution for each score triplet is specified by a 3 × 3 covariance matrix. Let us denote the covariance (equivalent to correlation) matrix for the *z*-value triplet (*z*_*g*_, *z*_*g*′_, *z*_*g*′′_) by

(15) $$\begin{array}{cc} R_{z}^{\left[g,g^{\prime },g^{\prime \prime }\right]} & =E\left[\begin{array}{c} z_{g}\\ z_{g\prime }\\ z_{g^{\prime \prime }} \end{array}\right]\left[\begin{array}{ccc} z_{g} & z_{g^{\prime }} & z_{g^{\prime \prime }} \end{array}\right]\\ & =\left[\begin{array}{ccc} 1 & \rho \left(z_{g},z_{g^{\prime }}\right) & \rho \left(z_{g},z_{g^{\prime \prime }}\right)\\ \rho \left(z_{g^{\prime }},z_{g}\right) & 1 & \rho \left(z_{g^{\prime }},z_{g^{\prime \prime }}\right)\\ \rho \left(z_{g^{\prime \prime }},z_{g}\right) & \rho \left(z_{g^{\prime \prime }},z_{g^{\prime }}\right) & 1 \end{array}\right]. \end{array}$$

For each *z*-score triplet, **R**_*z*_ is an element of the space—call it *ℛ* ^3^—of all symmetric positive-semidefinite matrices in ℝ^3  ×  3^ with element magnitudes no greater than unity. **R**_*z*_ is continuously distributed over *ℛ* ^3^.

Again, we need an empirical way to compute the joint moments of the *z* scores. Let *q*_**R**_*z*__(Ξ) be the empirical density of the $\left(\begin{array}{c} G\\ 3 \end{array}\right)$ correlation matrices, **R**_*z*_. This density must be inferred from the observed data. Of practical importance is the fact that, although each **R**_*z*_ is distributed over a subspace of ℝ^3  ×  3^, the domain of *q*_**R**_*z*__ is effectively a three-dimensional manifold of that space because each covariance matrix is unambiguously determined by its three values above or below its main diagonal (with unity diagonal elements). The argument Ξ may be thought of as a vector of these three elements (over the continuum of allowable values), but we will continue to denote it as a matrix as a reminder of its association with **R**_*z*_. We have, in conjunction with (6)–(14), the following useful approximation.

**Lemma 2 .**

The third-order joint central moment of a histogram count triplet (*Y*_*b*_, *Y*_*b*′_, *Y*_*b*′′_), where two or more indices may be equal, is approximated by

(16) $$\begin{array}{cc} & \hat{\gamma }\left(Y_{b},Y_{b^{\prime }},Y_{b^{\prime \prime }}\right)\\ & \;≝{\left[G!\right]}_{3}\;\Delta^{3}Q_{R_{z}}\left(z^{\left[b\right]},z^{\left[b^{\prime }\right]},z^{\left[b^{\prime \prime }\right]}\right)\\ & \;\;+{\left[G!\right]}_{2}\;\Delta^{2}[\delta_{bb^{\prime \prime }}Q_{\rho_{z}}\left(z^{\left[b\right]},z^{\left[b^{\prime \prime }\right]}\right)\\ & \;\;\;\;\;\;\;+\delta_{bb^{\prime }}Q_{\rho_{z}}\left(z^{\left[b\right]},z^{\left[b^{\prime }\right]}\right)\\ & \;\;\;\;\;\;\;+\delta_{b^{\prime }b^{\prime \prime }}Q_{\rho_{z}}\left(z^{\left[b^{\prime }\right]},z^{\left[b^{\prime \prime }\right]}\right)]\\ & \;\;+\delta_{bb^{\prime }b^{\prime \prime }}\hat{\mu }\left(Y_{b}\right)-\hat{\mu }\left(Y_{b}\right)\hat{\mu }\left(Y_{b^{\prime }}\right)\hat{\mu }\left(Y_{b^{\prime \prime }}\right)\\ & \;\;-[\hat{\mu }\left(Y_{b}\right)\hat{\varphi }\left(Y_{b^{\prime }},Y_{b^{\prime \prime }}\right)+\hat{\mu }\left(Y_{b^{\prime }}\right)\hat{\varphi }\left(Y_{b},Y_{b^{\prime \prime }}\right)\\ & \;\;\;\;+\hat{\mu }\left(Y_{b^{\prime \prime }}\right)\hat{\varphi }\left(Y_{b},Y_{b^{\prime }}\right)], \end{array}$$

where

(17) $$\begin{array}{cc} & Q_{R_{z}}\left(\zeta_{1},\zeta_{2},\zeta_{3}\right)\\ & \;=\overset{}{\underset{R^{3}}{\int }}\frac{q_{R_{z}}\left(\Xi \right)}{{\left(2\pi \right)}^{3/2}{\left|\Xi \right|}^{1/2}}\\ & \;\;\times \exp\{\;-\frac{1}{2}\left[\begin{array}{ccc} \zeta_{1} & \zeta_{2} & \zeta_{3} \end{array}\right]\Xi^{-1}{\left[\begin{array}{ccc} \zeta_{1} & \zeta_{2} & \zeta_{3} \end{array}\right]}^{T}\}\text{d}\Xi , \end{array}$$

$\hat{\mu }$, *Q*_*ρ*_*z*__, $\hat{\varphi }$, and [*G*!]_*ℓ*_ are defined in (6) and (11), and Δ is the *z*-histogram bin width.

To obtain the moments of *p*(*𝔉*, *C*) it is simply necessary to combine the moments of the corresponding *Y*_*b*_ counts. For example,

(18) $$\begin{array}{cc} \sigma_{F}^{2} & =E\left\{{\left(F-\mu_{F}\right)}^{2}\right\}\approx \underset{\left\{b,\;b^{\prime }:\;Z_{b},Z_{b^{\prime }}\subset Z_{C}\right\}}{\sum }\hat{\varphi }\left(Y_{b},Y_{b^{\prime }}\right),\\ \sigma_{C}^{2} & =E\left\{{\left(C-\mu_{C}\right)}^{2}\right\}\approx \underset{\left\{b,\;b^{\prime }:\;Z_{b},Z_{b^{\prime }}\subset Z_{C}\right\}}{\sum }\hat{\varphi }\left(Y_{b},Y_{b^{\prime }}\right),\\ \varphi \left(C,F\right) & =E\left\{{\left(C-\mu_{C}\right)}^{2}{\left(F-\mu_{F}\right)}^{2}\right\}\\ & \approx \underset{\left\{b,\;b^{\prime }:\;Z_{b}\subset Z_{F},\;Z_{b^{\prime }}\subset Z_{C}\right\}}{\sum }\hat{\varphi }\left(Y_{b},Y_{b^{\prime }}\right). \end{array}$$

The key quantities in the lemmas for approximating moments are the empirical covariance densities. Obtaining these densities in the presence of severe sampling errors is discussed next.

### 4.2. Empirical Correlation Densities

#### 4.2.1. Approach

 Because of severe sampling fluctuations, the current methods can recover only *q*_*ρ*_*z*__(*ξ*) from the data. This density is then used to estimate *q*_**R**_*z*__(Ξ). For this purpose, as well as to facilitate the calculations of Lemmas 1 and 2, we seek to parameterize the requisite densities. For most real examples, a single omnibus parameter *α* is found to be sufficient.

#### 4.2.2. Data Normalization

For all-null false discovery rate calculations, normalization of the per-microarray expression results has been found to be beneficial [31, Remark E]. The columns of the data matrix **X** are standardized to mean zero and unity variance. This standardization normalizes output “brightness” among microarrays [53, 54]. It also forces the sum of covariances, and approximately the sum of correlations, to be zero. This permits the fitting of a zero symmetric density to *q*_*ρ*_*z*__(*ξ*), which, in turn, has profound consequences for the form of *q*_**R**__*z*_(Ξ).

 Formally, let **X** ^*o*^ denote the *residual expression matrix*, obtained by subtracting from **X** each gene's average response within each treatment group, and let *x*_*g*•_^*o*^ denote the marginal random variable modeling the residual expression outcomes for gene *g* [like *x*_*g*•_ of Assumption (4), p. 5]. All further discussion of gene expression values will refer to these normalized residual values.

#### 4.2.3. Obtaining *q*_*ρ*_*z*__(*ξ*)

 The empirical densities *q*_*ρ*_*z*__ and *q*_**R**_*z*__, as well as others to be introduced below, clearly play a key role in moment estimation above. In each case, the empirical density—a surrogate for the true statistical density of the correlation coefficient(s) being modeled—is a distribution of a correlation function or matrix over a continuum, but it must be inferred from the data samples.

To deduce *q*_*ρ*_*z*__(*ξ*), we require *q*_*ρ*_*x*__(*ξ*)—the empirical density of the $\left(\begin{array}{c} G\\ 2 \end{array}\right)$ *gene expression* correlation coefficients. The mapping between the domains of *q*_*ρ*_z__ and *q*_*ρ*_*x*__ is needed, in principle, to calibrate *q*_*ρ*_z__. However, for the usual two-sample *t*-statistic, assuming independent columns in **X** ^*o*^, *ρ*(*z*_*g*_, *z*_*g*′_) ≈ *ρ*(*x*_*g*•_, *x*_*g*′•_) [recall Assumption (4), p. 5]; hence, *q*_*ρ*_*z*__(*ξ*) ≈ *q*_*ρ*_*x*__(*ξ*). We make the assumption of equality of these densities below.

Let $\overset{̅}{\varphi \;}{(x}_{g•}^{o},x_{g^{\prime }•}^{o})$ denote the *sample covariance* between rows (genes) *g* and *g*′ of **X** ^*o*^. For convenience, we define the notation

(19) $$\begin{array}{c} {\bar{\rho }}_{gg\prime }≝\bar{\rho }\left(x_{g•}^{o},x_{g^{\prime }•}^{o}\right)=\frac{\overset{̅}{\varphi }\left(x_{g•}^{o},x_{g^{\prime }•}^{o}\right)}{\;\sqrt{\overset{̅}{\varphi }\left(x_{g•}^{o},x_{g•}^{o}\right)\overset{̅}{\varphi }\left(x_{g^{\prime }•}^{o},x_{g^{\prime }•}^{o}\right)}}. \end{array}$$

These are the values to be fit with density *q*_*ρ*_*x*__(*ξ*).

To reduce the variability added by sampling errors, we apply the Fisher transform:

(20) $$\begin{array}{c} {\bar{\tau }}_{gg^{\prime }}=\frac{1}{2}\log\frac{1+{\bar{\rho }}_{gg^{\prime }}}{1-{\bar{\rho }}_{gg^{\prime }}}. \end{array}$$

For bivariate normal samples, the Fisher transform has remarkable normalizing and variance stabilizing properties [55], and each ${\bar{\tau }}_{gg^{\prime }}$ is well modeled by the distribution ${\bar{\tau }}_{gg^{\prime }}\sim 𝒢(\tau_{gg^{\prime }},{\left[G-3\right]}^{-1})$, where *τ*_*gg*′_ is the Fisher-transformed underlying correlation coefficient. Assuming a sampling model

(21) $$\begin{array}{c} {\bar{\tau }}_{gg^{\prime }}=\tau_{gg^{\prime }}+ɛ;\;\;\tau_{gg^{\prime }}\sim p_{\tau }\left(\xi \right), \end{array}$$

where *p*_*τ*_ is the distribution of the Fisher-transformed underlying correlation coefficients, we can interpret the histogram of Fisher-transformed sample correlations, say the “${\bar{\tau }}_{gg^{\prime }}$-histogram,” as a convolution of *p*_*τ*_(*ξ*), the statistical correlation density on the scale resulting from the Fisher transform, and the histogram of sampling errors, say, the “*ɛ*-histogram,” also on the *τ*-scale. Then, the underlying *p*_*τ*_(*ξ*) is obtained by deconvolving this density from the convolved pair, $p_{\;\bar{\tau }\;}(\xi )=p_{\tau }(\xi )*p_{ɛ}(\xi )$. For a wide variety of microarray data sets studied in this work (also see [30]), the normal distribution *𝒢*(0, *σ* ^2^) fits nicely to the ${\bar{\tau }}_{gg^{\prime }}$ histogram. For bivariate normal samples, where *ɛ* ~ *𝒢*(0, [*G* − 3]^−1^), the estimate of *p*_*τ*_(*ξ*), say ${\hat{p}}_{\tau }(\xi )$, takes the normal form *𝒢*(0, *σ* ^2^ − [*G* − 3]^−1^). It is this estimate that will serve as the empirical density of the Fisher-transformed correlations, $q_{\tau }\equiv {\hat{p}}_{\tau }$.

Having obtained the underlying *q*_*τ*_(*ξ*), we must, in principle, undo the mapping (20) to obtain *q*_*ρ*_*x*__(*ξ*), then deduce *q*_*ρ*_*z*__ from *q*_*ρ*_*x*__. Recall, however, that we assume that the correlation coefficients of the *z* and *x* ^*o*^ variables are identical [31], so that we may directly seek *q*_*ρ*_*z*__(*ξ*) = *q*_*ρ*_*x*__(*ξ*) from *q*_*τ*_(*ξ*). A distribution that fits the inverse-transformed *q*_*τ*_(*ξ*) well is

(22) $$\begin{array}{cc} q_{\rho_{z}}\left(\xi \right)\propto {\left(1-\xi^{2}\right)}^{a} & ={\left[\frac{1}{2}\left(\xi +1\right)\right]}^{\alpha }{\left[1-\frac{1}{2}\left(\xi +1\right)\right]}^{\alpha },\\ & \;\;\;\;\;\;\;\;\;\;\;\;\;\;\left|\xi \right|\leq 1, \end{array}$$

a class of densities in the general *Beta* distribution family given by:

(23) $$\begin{array}{cc} p_{B}\left(\xi ;\alpha ,\beta \right) & =\frac{1}{B\left(\alpha ,\beta \right)}\xi^{\alpha -1}{\left(1-\xi \right)}^{\beta -1},\;\;\\ & \;\;\;\;\;\;\;\;\;0\leq \xi \leq 1, \end{array}$$

where *B* is the *Beta* function and *α* and *β* are nonnegative shape parameters. Comparing (22) and (23) gives a useful probabilistic interpretation of the correlation coefficient, say ${\tilde{\rho }}_{z}$, modeled by the empirical density *q*_*ρ*_*z*__: We see from (22) that ${\tilde{\rho }}_{z}\sim p_{B}(0.5\xi +1;\alpha ,\alpha )$. Therefore, $\sigma_{{\tilde{\rho }}_{z}}^{2}$ will be a factor of four greater than the variance of a *Beta*-distributed random variable with parameters *α* = *β*. That is,

(24) $$\begin{array}{c} \sigma_{{\tilde{\rho }}_{z}}^{2}=4\frac{\alpha^{2}}{{\left(2\alpha \right)}^{2}\left(2\alpha +1\right)}⟹\alpha =\frac{1-\sigma_{{\tilde{\rho }}_{z}}^{2}}{2\sigma_{{\tilde{\rho }}_{z}}^{2}}. \end{array}$$

Thus, using $\sigma_{{\overset{̅}{\rho }}_{z}}^{2}$ as an estimate of $\sigma_{{\tilde{\rho }}_{z}}^{2}$, we obtain the parameter *α*, hence, the distribution *q*_*ρ*_*z*__.

#### 4.2.4. Obtaining *q*_*R*_*x*__(Ξ)

 We now pursue *q*_**R**_*x*__(Ξ) as an extension of *q*_*ρ*_*x*__(*ξ*). Like *q*_**R**_*z*__, the effective domain of *q*_**R**_*x*__ is of only three dimensions. Also similarly to the scalar density, for the two-sample *t*-statistic, *q*_**R**_*z*__(Ξ) ≈ *q*_**R**_*x*__(Ξ). Hence, we can pursue *q*_**R**_*z*__ indirectly by finding *q*_**R**_*x*__.

We seek a joint distribution on the space of all 3 × 3 correlation matrices such that all the inherent marginal distributions (i.e., the distributions of *ρ*(*x*_*g*•_^*o*^, *x*_*g*′•_^*o*^) for *g* ≠ *g*′) are equivalent to *q*_*ρ*_*x*__(*ξ*). Such a result can be obtained from the inverse-Wishart distribution whose marginalization properties are helpful when studying a subset of variables [56]. Suppose that the true statistical covariance matrix of **X** ^*o*^, say,

(25) ΣXo≝E{Xo(Xo)T}∈RG⊂RG×G     (for  simplicity  Σo≝ΣXo)

follows the inverse-Wishart distribution *𝒲*_*G*_^−1^(**I**, *ν*), *ν* ≥ *G*,

(26) $$\begin{array}{cc} & {\text{Σ}}^{o}\sim p_{{\;}_{{\text{Σ}}^{o}}}\left(\Xi \;|\;\nu \geq G\right)\propto {\left|\Xi \right|}^{-0.5(\nu +G+1)}\\ & \;\;\;\;\times \exp\left\{-0.5\;\;|\;\mathrm{tr}\left\{\Xi^{-1}\right\}\right\},\;\;\Xi \in R^{G\times G}, \end{array}$$

where *ν* is the single parameter that characterizes the distribution, and tr {·} indicates the trace. The goal is to relate *ν* to parameter *α* of (22) and to determine the distribution of any of the 3 × 3 covariance submatrices of Σ^*o*^.

Following the *separation strategy* of Barnard et al. [57], we decompose Σ^*o*^ into its variances and normalized covariances (i.e., correlation coefficients) as

(27) $$\begin{array}{c} {\text{Σ}}^{o}=SR^{o}S, \end{array}$$

where **S** ∈ ℝ^*G*×*G*^ is the diagonal matrix whose *i*th diagonal element, *s*_*i*_, is the standard deviation of the gene *i* residual expression [recall (2)]. **R** ^*o*^≝**R**_**X**_*o*__ is the *G* × *G* correlation matrix of the residual expression matrix **X** ^*o*^. Under the transformation Σ^*o*^ → (**S**, **R** ^*o*^), the Jacobian is given by (2∏_*i*_ *s*_*i*_)^*G*^ [58, Theorem 3]. Thus, after marginalization over **S**:

(28) $$\begin{array}{c} R^{o}\sim p_{R^{o}}\left(\Xi \;|\;\nu \right)\propto {\left|\Xi \right|}^{-0.5(\nu +G+1)}\overset{G}{\underset{i=1}{\prod }}\overset{\infty }{\underset{0}{\int }}s_{i}^{-(\nu +1)}e^{-\xi^{ii}/2s_{i}^{2}}ds_{i}, \end{array}$$

where *ξ* ^*ii*^ is the *i*th diagonal element of Ξ^−1^. The product arises because of independence of the *s*_*i*_ elements. Substituting *ω*_*i*_ = *ξ* ^*ii*^/2*s*_*i*_^2^ yields

(29) $$\begin{array}{cc} & R^{o}\sim p_{R^{o}}\left(\Xi \;|\;\nu \right)\;\propto {\left|\Xi \right|}^{-0.5(\nu +G+1)}{\left(\overset{}{\underset{i}{\prod }}\xi^{ii}\right)}^{-0.5\nu }\\ & \;\;\;\;\;\;\;\;\times \left(\overset{}{\underset{i}{\prod }}\overset{\infty }{\underset{0}{\int }}\omega_{i}^{0.5(\nu -2)}e^{-\omega_{i}}d\omega_{i}\right), \end{array}$$

which leads to an expression for the probability density of the matrix **R** ^*o*^:

(30) $$\begin{array}{c} p_{R^{o}}\left(\Xi \;|\;\nu \right)\;\propto {\left|\Xi \right|}^{0.5(\nu -1)(G-1)-1}{\left(\underset{i}{\prod }\left|{\left(\Xi \right)}_{ii}\right|\right)}^{-0.5\nu }, \end{array}$$

where (**A**)_*ii*_ denotes the *i*th principal submatrix of **A**, and where we have used the fact that *ξ* ^*ii*^ = |(Ξ)_*ii*_ | /|Ξ|. For **R** ^*o*^ with probability density (30), the marginal density of its arbitrary correlation submatrix also has a useful expression.

**Lemma 3 .**

For a correlation matrix **R** ^*o*^ ∈ *ℛ* ^*G*^ with the probability density (30), the *κ* × *κ* correlation submatrix, **R**_*κ*_^*o*^ ∈ *ℛ* ^*κ*^, has the density

(31) $$\begin{array}{cc} & {\;p_{R_{\kappa }^{o}}}_{{\;}_{\;}}\left(\Xi_{\kappa }\;|\;\nu \right)\propto {\left|\Xi_{\kappa }\right|}^{0.5(\nu -G+\kappa -1)(\kappa -1)-1}\\ & {\;\;\;\;\;\;\times \left(\overset{}{\underset{i}{\prod }}\left|{\left(\Xi_{\kappa }\right)}_{ii}\right|\right)}^{-0.5(\nu -G+\kappa )},\;\;\Xi_{\kappa }\in R^{\kappa }. \end{array}$$

**Proof**

Suppose that the *κ* × *κ* statistical covariance submatrix Σ_*κ*_^*o*^ undergoes the transformation Σ_*κ*_^*o*^ → (**S**_*κ*_, **R**_*κ*_^*o*^), where **S**_*κ*_ is the diagonal scaling matrix of appropriate standard deviations [recall (27)]. Then due to the marginalization property of inverse-Wishart, **R**_*κ*_^*o*^ ~ *𝒲*_*κ*_^−1^(**I**, *ν* − *G* + *κ*). Following steps(26)–(30) for Σ_*κ*_^*o*^ yields (31).

Substituting *κ* = 2 in result (31) yields

(32) $$\begin{array}{cc} p_{R_{2}^{o}}\left(\Xi_{2}\;|\;\nu \right) & \equiv p_{\rho_{12_{\;}}}\left(\xi \;|\;\nu \geq G\right)\propto {\left(1-\xi^{2}\right)}^{0.5(\nu -G-1)\;},\\ & \;\;\;\;\;\;\;\;\;\;\;\;\;\;\;\;\left|\xi \right|\leq 1. \end{array}$$

Note that this density is the function of a scalar argument, the single value of the off-diagonal elements of **R**_2_^*o*^. We have indicated this by use of the subscript “*ρ*_12_” in the second term in the expression. The critical property of this result is that it has the same uniparametric form as (22). By setting *ν* − *G* = 2*α* + 1 we can force the inherent marginal densities of the **R** ^*o*^ entries (*ρ*(*x*_*g*•_^*o*^, *x*_*g*′•_^*o*^), *g* ≠ *g*′) to equal *q*_*ρ*_*x*__(*ξ*)—the specific aim of this derivation.

Finally, substituting *κ* = 3 in result (31), we obtain

(33) $$\begin{array}{c} q_{R_{x}}\left(\Xi \right)\propto \frac{{\left(1-\xi_{12}^{\;2}-\xi_{23}^{\;2}-\xi_{13}^{\;2}+2\xi_{12\;}\xi_{23\;}\xi_{13}\right)}^{2(\alpha +1)}}{{\left[\left(1-\xi_{12}^{\;2}\right)\left(1-\xi_{23}^{\;2}\right)\left(1-\xi_{13}^{\;2}\right)\right]}^{\alpha +2}}. \end{array}$$

in which *ξ*_*ij*_ is the (*i*, *j*) element of the evaluated matrix (in the abstract) Ξ. The density of a particular covariance matrix in *ℛ* ^3^, say Ξ = **R**_*x*_, involves the use of the three elements in the upper triangle of the matrix, reinforcing earlier assertions that the domain of *q*_**R**_*x*__ is a manifold of the matrix space. Recall that **R**_*x*_^[*g*,*g*′,*g*′′]^ (assumed equivalent to **R**_*z*_^[*g*,*g*′,*g*′′]^) is the original notation for the 3 × 3 covariance matrix of a score triplet (*z*_*g*_, *z*_*g*′_, *z*_*g*′′_), and, by extension, (*x*_*g*•_^*o*^, *x*_*g*′•_^*o*^, *x*_*g*′′•_^*o*^). In the present discussion, **R**_*x*_ assumes the role of a 3 × 3 submatrix of **R** ^*o*^, namely, **R**_3_^*o*^.

For large *G*, the assumption of the inverse-Wishart distribution in (26) is not well justified. However, the assumption is used here strictly for its value in deducing *q*_**R**_*x*__(Ξ) from *q*_*ρ*_*x*__(*ξ*). There is no concern for a model of the entire matrix **R** ^*o*^ = **R**_**X**^*o*^_. Further, single-parameter distributions on a positive definite matrix space are few. The inverse-Wishart distribution is chosen for its useful marginalization property. Of course, a tenuous assumption is not justified by a useful property if it leads to an unuseful procedure. The practical validation of the assumption is manifest in Section 5. The “Bayesian correlation priors” point of view from the work of Liechty et al. [59] was especially helpful in formulating these ideas. Exploring other ways to obtain *q*_**R**_*x*__ is a worthwhile but challenging endeavor.

Finally, we have derived *p*_**R**_^*o*^_*κ*_(Ξ_*κ*_ | *ν*) only up to a proportionality constant. However, for *q*_**R**_*x*__(Ξ)(*κ* = 3), normalization is straightforward. For *κ* ≥ 4 it is necessary to resort to Monte Carlo methods which only require densities up to a proportionality constant.

## 5. Results

### 5.1. Testing on Real Data Sets

A MATLAB implementation of the approach based on the work above is available at the website http://www.egr.msu.edu/~deller/. The methods were tested on two real data sets, both showing a significant amount of intergene covariance but exactly opposite *𝔉* behaviors. Calculations below are for left-sided tail-area parameter *δ* = −2.5, and center area parameter *c* = 1 [recall (4)]. Comparisons with Efron's [31] second-order estimator of *𝔉* are made.

The first data set is from the breast cancer (BRCA) study of Hedenfalk et al. [60]. These data record the expression of *G* = 3226 genes on *M* = 15 microarrays with seven samples assigned to BRCA1 mutations and eight to BRCA2. The original research seeks to identify genuine mRNA activity differences between these two categories. In the present paper, the logarithm is applied to the mRNA levels to increase Gaussianity [61].

In a study of the human immunodeficiency virus (HIV), Van 't Wout et al. [62] investigated *G* = 7680 genes over *M* = 8 microarrays with four samples assigned to an HIV infected condition and the remaining four to the control. To produce the test cells, the control cells (CD4 T cell lines) were infected by the HIV − 1_BRU_ virus. The paper reports raw mRNA levels which, like the BRCA data, are converted to logarithms in the present work.

**Figure 1:**
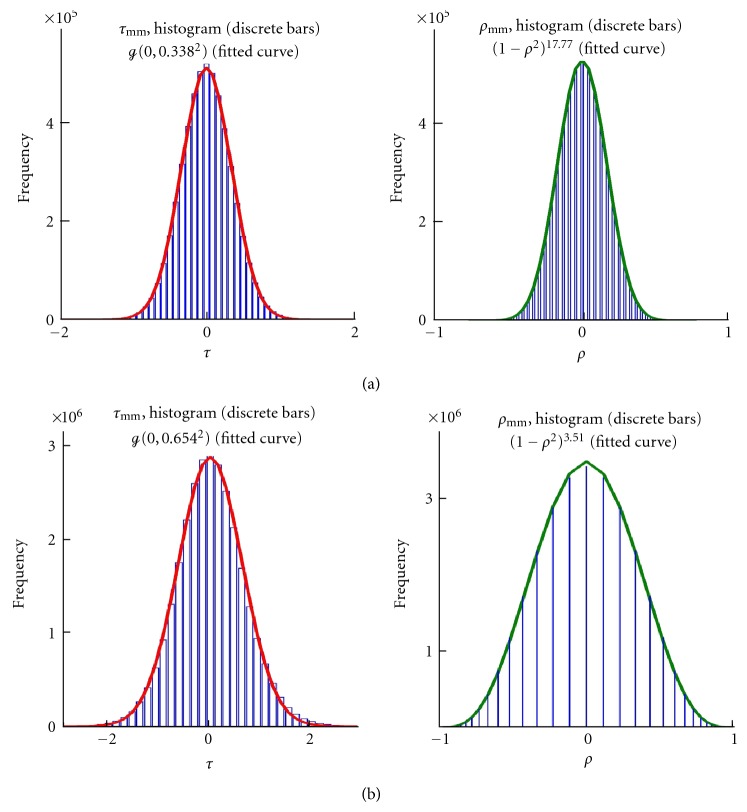
Effect of sampling fluctuations on the empirical covariance density. (a) Upper curves: BRCA data. (b) Lower curves: HIV data. For each subfigure: left panel is the histogram of sample covariances after applying the Fisher transformation (20) and a normal distribution (heavy curve) fitted to it; right panel is the histogram of denoised covariances and a modified beta distribution fitted to it (heavy curve). These distributions summarize the cumulative effect of (_2_^*G*^) gene-gene covariances in a single parameter (*α*) distribution.

The present analysis reduces an entire expression matrix to two numbers: the center area null count, *C*, and the omnibus parameter, *α*. As is evident in Figure 1, the parametrization described in Section 4 is realistic. For the BRCA data, *α* = 17.77, and for HIV, *α* = 3.51. The Figure 1 caption provides details.

**Figure 2:**
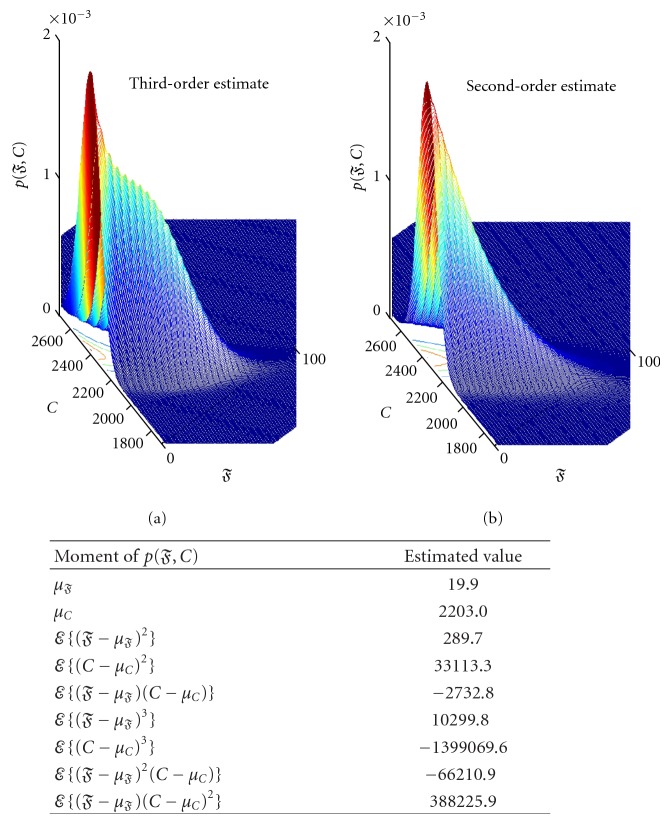
BRCA example: estimated *p*(*𝔉*, *C*) moments and estimated distributions using maxent, $\hat{p}(𝔉,C)$. The distribution estimate on the left uses third-moment information in the maxent optimization, while the right estimate uses only second moments. The third-order estimate exhibits finer details than its second-order counterpart, and a contour that cannot be modeled using a quadratic distribution.

The next step is to compute the moments of *p*(*𝔉*, *C*) per Lemmas 1 and 2. These calculations require parameters *G*, *α*, *c*, *δ*, and Δ. We set Δ = 0.1. These estimated moments are used to find the maximum-entropy (maxent) distribution $\hat{p}(𝔉,C)$ (Appendix B).  Figure 2, for example, reports the moments and the corresponding maxent distribution for the BRCA data. *𝔉* and *C* exhibit strong negative correlation of −0.89, a value similar to that in [31, Table 1]. Furthermore, *𝔉* shows significant positive skewness, which causes *C* to exhibit negative skewness. This is not surprising as *𝔉* is bounded below by zero, and yet has small mean but inflated variance. The third-moment provides an additional level of detail about the joint behavior of *𝔉* and *C*.

During the maxent numerical optimization, a 100 × 500 equispaced mesh was found sufficient for the BRCA data; however, for the HIV data the resolution had to be increased to 400 × 2000. This is because, in addition to the larger *G*, the HIV **X** also exhibits more covariance. The BRCA optimization required 30 iterations, while HIV took ~70 iterations, to converge to an estimated distribution.

**Figure 3:**
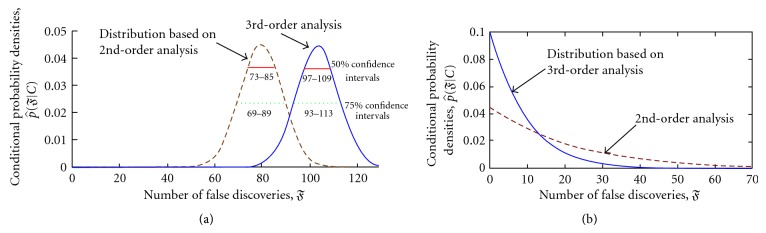
Estimated conditional distributions of the number of false discoveries $\hat{p}(𝔉|C)$. Panel (a) BRCA data. Panel (b) HIV data. To show the effect of skewness corrections the third-moment *𝔉* distribution (solid curve) is compared to its second-moment counterpart (dashed curve). For BRCA the second-moment mean estimate is 79 compared to 104 for the third-moment, while for HIV these are 19 and 8. The BRCA curves are also labeled with 50% (solid line) and 75% (dotted line) confidence intervals.

Figure 3 reports the estimated *p*(*𝔉*∣*𝒪*), where *𝒪* refers to the observed data. Second- and third-moment estimates are shown separately. In the framework of statistical inference such a distribution is the ultimate goal, but this result could later be used for other purposes like computing point estimates and associated confidence intervals.

If the mean of the estimated $\hat{p}(𝔉|𝒪)$ is used as a point estimate of *𝔉*, then for the BRCA data, third-moment calculations suggest 104 false discoveries versus 79 for the second-moment while the usual ${\hat{\mu }}_{𝔉}\approx ℰ\{𝔉\}$ suggests only 20 false discoveries. These numbers must be put in perspective by noting that the actual *z*_*g*_ count falling in the left-sided tail-area is 116. For the HIV data, the third-moment analysis found eight false discoveries compared to 19 for second-moment, while the mean estimator ${\hat{\mu }}_{𝔉}\approx ℰ\{𝔉\}$ produces 48. This time the *z*_*g*_ count in the left-sided tail area is 46. Clearly, extensive analysis of intergene dependence can lead to very different conclusions from the same data, relative to those of the mean estimate of false discoveries (and, in turn, the procedures built around it).

The second-order estimator designed by Efron [31] found 77 false discoveries for BRCA. Efron compares that to the results of nonparametric analysis in the same paper and concludes underestimation, but the issue is not further pursued. Our findings show that nonlinear dependence (as reflected in the present case by moments higher than second) is potentially very important in characterizing the null *z* histogram.

We note in passing that the availability of $\hat{p}(𝔉|𝒪)$ permits the application of the bound *ℙ*(*𝔉*/*G*_∗_∣*𝒪* ≥ *γ*) ≤ *λ* as a control measure, as recommended by Lehmann and Romano [63]. It is not a trivial matter to choose *γ* and *λ* such that a fair comparison with other error measures is possible; however, for illustrative purposes, we set *γ* = 0.15 and *λ* = 0.5. With this constraint, the present approach reports 174 discoveries (108 for second-moment) for the HIV **X**. This compares favorably with the results of Efron [31], where the Benjamini-Hochberg procedure, with false discover rate control level 0.10 and an empirical null from [5], yields 180 discoveries. Without covariance modeling, the Benjamini-Hochberg procedure reports only 20 discoveries.

### 5.2. Testing on Simulated Data

Insight is gained by testing the approach on simulated data for which the “correct answer” is known. In the studies below, all cases are null (no treatment, residuals only). The goal is to see how well the realized left-sided tail-area count can be estimated from the center count. To maintain realism, we simulate raw mRNA levels. The testing scenario is a “two-group study,” so en route to *z*-values we take the usual two-sample *t*-statistic.

Let the mRNA expression level, *x*_*gm*_, of gene *g* measured by microarray *m*, be distributed as the *Gamma density*: For *m* = 1,…, *M*,

(34) $$\begin{array}{c} x_{gm}\sim {p_{\Gamma }}_{\;}\left(\xi ;\kappa ,\theta \right)=\xi^{\kappa -1}\frac{e^{-\xi /\theta }}{\theta^{\kappa }\Gamma \left(\kappa \right)},\;\xi \geq 0,\;\kappa ,\theta >0, \end{array}$$

where Γ(*κ*) = ∫_0_^*∞*^ *v* ^*κ*−1^ *e* ^−*v*^ *dv* is the Gamma function. *κ* and *θ* are called the *shape parameter* and the *scale parameter* of the distribution, respectively. This distribution is similar to the Gamma-Gamma model used by Newton et al. [64]. In (34) the shape parameter *κ* is common to all genes, while the scale parameters {*θ*_*g*_}_*g*=1_^*G*^ characterize varying mRNA levels from gene to gene, but are assumed i.i.d. as

(35) $$\theta_{g}\overset{\text{i.i.d.}}{\sim }p_{\Gamma }\left(\xi ;\kappa_{0},\theta_{0}\right),\;\text{for}\;g=1,\ldots ,G.\;$$

The intuition that genes with larger underlying mRNA levels would have higher variance is supported by model (34) since the mean of the *g*th gene is *κθ*_*g*_ and variance is *κθ*_*g*_^2^.

The parameter set (*κ*, *κ*_0_, *θ*_0_) in (34) and (35) can be chosen on the basis of the overall gene expression histogram of real microarray data. Results for three such parameter sets: (1,0.6,500), (2,0.39,384), and (3,0.33,300), are presented. These numbers were chosen to preserve the total sample variance, and the particular values are based on the HIV data of Van 't Wout et al. [62] which were collected using Affymetrix microarrays. In particular, *κ* = 1 models *x*_*gm*_ variables that are exponentially distributed, *κ* = 2 models a unimodal distribution with heavy tails and a noticeable departure from Gaussianity. Case *κ* = 3 represents an approximation to a Gaussian distribution, but with slightly heavier tails.

Substantial row-wise covariance was added via the Gaussian copula technique: A (*G* × *M*) matrix, say **Z**, of i.i.d. unit normal RVs was used to produce a correlated matrix, **Z** ^*c*^, via the mapping

(36) $$\begin{array}{c} Z^{c}=L^{T}Z,\;\;\text{where}\;Z\;\text{has}\;\text{elements}\;z_{gm}\overset{\text{i.i.d.}}{\sim }G\left(0,1\right), \end{array}$$

in which **L** is the lower-triangular Cholesky factor (e.g., [65]) of **R**_*z*,*G*_ + *ɛ* **I**_*G*_, the correlation matrix of the actual expression matrix **X** from the BRCA study, plus a small diagonal load to prevent singularity due to the fact that *M* < *G*. Several other dense matrices, **R**, generated through a different method [66], yielded similar results. This process imposes the covariance of the real BRCA data on the simulated substrate of independent Gaussian variables. The resulting elements *z*_*gm*_^*c*^ were mapped to *P* values, *P*_*𝒢*_*u*__(*z*_*gm*_^*c*^), then further transformed to simulated expression variables, *x*_*gm*_, through the inverse Gamma c.d.f. as in (34). The result is the simulated expression matrix **X** = [*x*_*gm*_]_*G*×*M*_.

**Figure 4:**
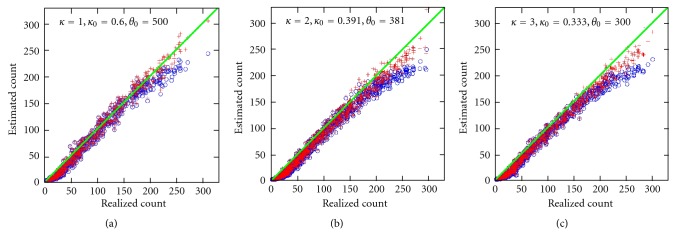
Simulation experiments comparing conditional estimates: third-moment estimates (+ marker) and second-moment (∘ marker). This figure corresponds to the left-sided tail area with *δ* = −2.0 [see (4)]. Substantial rowwise covariance is present. The abscissa is the realized count while the ordinate is the estimated count. The significance of parameters (*κ*, *κ*_0_, *θ*_0_) is discussed the text. Third-moment skewness corrections tend to make the estimation process more accurate.

**Figure 5:**
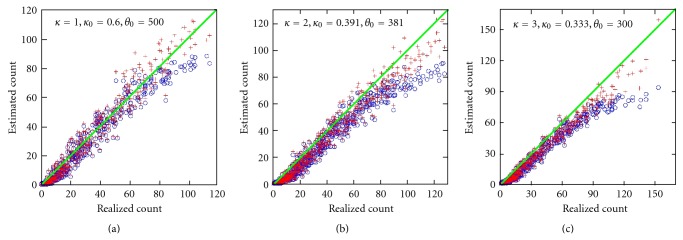
Left-sided tail area with *δ* = −2.5. Otherwise Figure 4 caption is applicable.

Figures 4 and 5 compare second- and third-moment estimates for *δ* = −2.0 and *δ* = −2.5, respectively. In both cases, *c* = 1 for the center area (see Section 6). For each (*κ*, *κ*_0_, *θ*_0_), 800 matrices **X** were processed. On each **X** the approach was applied in its entirety and no additional knowledge was assumed. The *a posteriori* mean was used as the final estimate. The usual mean estimator ${\hat{\mu }}_{𝔉}\approx ℰ\{𝔉\}$ consistently reported 20 for *δ* = −2.5 and 73 for *δ* = −2.0, regardless of the particular **X**.

Strikingly, for all three parameter sets (*κ*, *κ*_0_, *θ*_0_), the third-moment skewness corrections make the estimation process more accurate. For some of the scenarios third-moment estimates saturate somewhat, but the effect is minor compared to that in the second-order approaches. To the extent that these parameter sets cover a wide range of realistic gene expression data, the practical utility of the proposed approach is evident.

## 6. Discussion

Advances in DNA microarray technology, improved standardization procedures, and a careful execution of laboratory protocols collectively lead to testing situations with marginally correct but strongly correlated null hypotheses. If correlation is the result of intrinsic gene-gene interactions, no experimental design can circumvent it. Correlation can cause the realized $\dot{𝔉}$ to vary significantly from case to case [63], and the control of $ℰ\{\dot{𝔉}\}$ via the usual *μ*_*𝔉*_ = *ℰ*{*𝔉*} may no longer represent the basic facts. The moment theory of the null statistic histogram can be used to deduce an estimator of *μ*_*𝔉*_ which explicitly combines identifiability and covariance. Though we have explored these ideas in the differential analysis context above, the findings are quite general.

It is reasonable to question the necessity of the heavy mathematical machinery of the foregoing sections since it is possible to simulate a number of sets of *z*-scores {*z*_*g*_}_*g*=1_^*G*^ from the distribution **z** ~ *𝒢*(0, Σ_*z*_^*G*  ×  *G*^), then estimate the moments. However, due to sampling fluctuations, the underlying covariance matrix Σ_*z*_^*G*×*G*^ is ordinarily unattainable; however, pursuing quantities like *q*_*ρ*_*z*__(*ξ*) and *q*_**R**_*z*__(**ξ**) is still possible. Also, as *G* gets larger (~25,000 for recent microarray studies) computational demands, as well as the large number of *z*-score sets required, become prohibitive.

Permutation calculations, as in [31, Section 4], offer an alternative way to estimate the moments. They too can run into computational difficulties, especially when the test statistic itself is computationally intensive. Further difficulty arises when samples are few. For a two-group study like HIV (four samples each condition), the data provide only 70 unique permutations.

When a direct extraction of inter-hypotheses covariance is not feasible, single omnibus parameter models remain useful in that the investigator can still use judgment to intelligently incorporate some form of covariance effect by setting a value of the parameter *α*.

**Figure 6:**
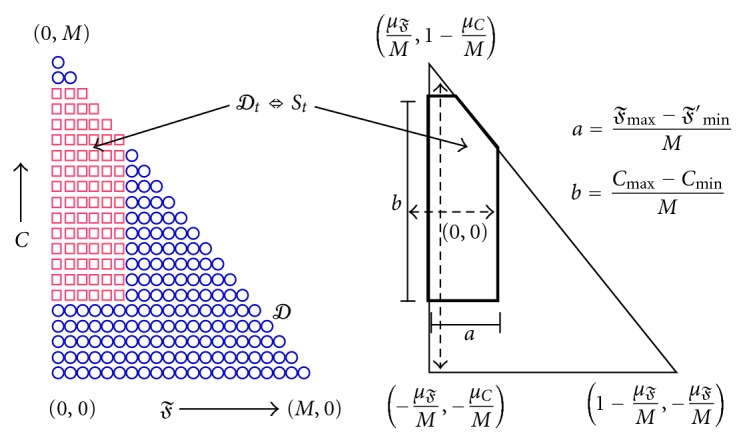
Discrete support *𝒟*_*t*_ (□ markers) versus continuous support *𝒮*_*t*_ (solid boundary). *𝒮*_*t*_ is normalized to improve numerical stability.

The distribution of interest *p*(*𝔉*, *C*) resides over support domain *𝒟* as shown in Figure 6, and the maxent algorithm is adept at handling such complicated support regions. At a more fundamental level, through maxent, we seek to minimize the amount of unintentional prior information brought into the inference.

Apart from the numerical parameters Δ (bin width) and the mesh resolution in maxent, the only open choice of parameterization in the present method is *c*, the center area boundary. The selection of *c* = 1 in this paper is based on the first eigenvector analysis of Efron [31] which suggests that (within certain approximations) the interval [−1,1] has completely opposite count behavior from the rest of the *𝒵* space.

One surprising result of this and similar studies is that more inter-*z*_*g*_ covariance does not translate into more extreme covariance between variables *𝔉* and *C*. In the BRCA data, for example, the coefficient between *𝔉* and *C* is −0.89, while for the HIV data the covariance drops to −0.75. Further research to gain insight into this behavior would be very useful.

Finally, while covariance was viewed in the present paper as a “destructive factor” in the attempt to estimate *𝔉*, inter-*z*_*g*_ covariance can, in fact, be *exploited* to increase power by finding a superior ranking of potential discoveries. The recent multiple testing literature has begun to address this possibility.

##  Ethical Approval

 Human subject data used in this study are publicly available and anonymous and are, therefore, exempted from continuing Internal Review Board scrutiny according to US Health and Human Services Policy 45 CFR 36, Subpart A, §46.101 (2.b.4).

## Figures and Tables

**Table 1:** Notation used for elementary scalar quantities. *v*, *v*__1__, and *v*__2__ are RVs and *ℰ* denotes the expectation.

Mean, average	*μ*_*x*_ or *μ*(*x*) = *ℰ*{*x*}	Standard deviation	$\sigma_{v}≝\sqrt{ℰ\left\{{\left(v-\mu_{v}\right)}^{2}\right\}}=\sqrt{\varphi (v,v)}$
Covariance	*φ*(*v*__1__, *v*__2__)≝*ℰ*{(*v*__1__ − *μ*_*v*__1___)(*v*__2__ − *μ*_*v*__2___)}	Variance	*σ*_*v*_^2^≝*ℰ*{(*v* − *μ*_*v*_)^2^} = *φ*(*v*, *v*)
Correlation	*ℰ*{*v*_1_, *v*_2_}≝*φ*(*v*_1_, *v*_2_) + *μ*_*v*_1__ *μ*_*v*_2__ (no special symbol reserved)	Correlation coefficient	$\rho \left(v_{1},v_{2}\right)≝\frac{\varphi \left(v_{1},v_{2}\right)}{\sigma_{v_{1}}\sigma_{v_{2}}}\;(\text{normalized}\;\text{covariance})$

## Appenddix

### A. Genes and Microarrays

To understand the core statistical methods developed in this paper, it is only necessary to know some very general facts about the biological application. The “blueprint” for building the components of every cell in an organism (except some viruses) is encoded in macromolecules collectively called *deoxyribonucleic acid* or “DNA.” Each DNA molecule consists of a complementary pair of long sequences (polymers) of small molecular elements known as *nucleotides*. The characteristic “double helix” configuration of DNA molecules results from chemical forces as the nucleotides on the complementary polymers strongly bind to one another. Each nucleotide is built around one of the four nitrogen bases guanine, adenine, thymine and cytosine, commonly known by their initial letters G, A, T, and C. It is the sequence of these bases that encodes the information for producing proteins that, in turn, determine the structures and functions of cells. In the human genome (the complete set of life-sustaining instructions in the genetic material of an organism), the DNA consists of about three billion nucleotides organized into 23 sets of structures called *chromosomes*.

*Genes* are certain identifiable sections of the DNA—varying in length from a few hundred to a few million bases—that encode a particular trait or “hereditary unit” of biological information by specifying and regulating the types of proteins produced. Almost every gene is present in the DNA of every cell of a given organism, but only a relatively few genes are active in any given cell type. Genes comprise only about 1% of the DNA material in the human geneome. The reasons for the existence of the remaining 99% of the DNA remain the subject of much conjecture, hypothesis, and research. It is currently estimated that there are on the order of 25,000 genes in the human genome, 99% of which are identical among all humans. The slight variances in the other 1% of the genes (gene *alleles*) determine differences among individuals (e.g., eye, hair, and skin color, blood type, etc.). Apart from normal variations (alleles), mutations in genes can lead to the formation of abnormal proteins with beneficial, neutral, or negative consequences for cell function and replication.

A gene is defined by its sequence of nucleotide bases, which, in turn, can be expressed as a sequence whose elements come from the set of four letters G, A, T, and C as described above—thus reducing the information carried by the gene to a simple code like “ATCGCT…”. A three letter code (i.e., a three base set like “AGA”), called a *codon*, ultimately specifies one of the 20 *amino acids* that are the building blocks of proteins. There are 64 possible codons, and 61 of these are used to indicate 20 amino acids, so there is redundant representation of the amino acids in the codons. The remaining three codons are used for regulating the protein synthesis.

The manifestation of genes as proteins is called *gene expression*. The degree to which a gene is “active” in a given collection of cells (e.g., a basal cell (skin) tumor) in a given set of conditions (e.g., untreated *versus* treated with radiation or chemotherapy) can be ascertained by measuring the levels of certain molecules [messenger RNA (mRNA) or complementary DNA (cDNA)] related to to proteins manufactured by the gene. A microarray consists of thousands of binding sites (“probes”), each populated with DNA fragments that can be associated with particular genes. The extent to which a gene is expressed in a particular preparation determines the extent to which the related microarray site “lights up” as the phosphorescent mRNA or cDNA in the preparation binds to its site. A single microarray experiment can be used to simultaneously quantify the expression levels of thousands of genes in a particular tissue preparation.

The applied purpose of the statistical modeling work in this paper is to develop methods for determining from microarray data which genes are expressed at significantly different levels when a cell type is exposed to different conditions. Ultimately, this information can be used to understand normal and abnormal cell function and replication, to target genes for medical therapies, and to develop drugs for treating myriad diseases and systemic disorders at the cellular level.

### B. Estimating *P* (*𝔉*, *C*) by Maximum Entropy Optimization (MAXENT)

#### B.1. MAXENT Distribution

*p*(*𝔉*, *C*) is a discrete distribution with support domain

(B.1) $$\begin{array}{c} D=\left\{\left(i,j\right):\;i,j\in Z;\;0\leq i\leq G,\;0\leq j\leq G,\;0\leq i+j\leq G\right\}. \end{array}$$

Any moment-based inference involves computation over *𝒟* whose increasing cardinality (∝*G* ^2^) makes processing difficult for a large *G*. However, computation can be reduced substantially by truncating the domain *𝒟* to the set

(B.2) $$\begin{array}{c} D_{t}=\{\left(i,j\right):F_{\min}\leq i\leq F_{\max},\\ \;C_{\min}\leq j\leq C_{\max},\;0\leq i+j\leq G\}, \end{array}$$

where,

(B.3) $$\begin{array}{cc} & F_{\min}=\max\left(\left\lfloor \mu_{F}-l\sigma_{F}\right\rfloor ,0\right),\;\\ & F_{\max}=\min\left(\left\lceil \mu_{F}+l\sigma_{F}\right\rceil ,G\right),\\ & C_{\min}=\max\left(\left\lfloor \mu_{C}-l\sigma_{C}\right\rfloor ,0\right),\;\\ & C_{\max}=\min\left(\left\lceil \mu_{C}+l\sigma_{C}\right\rceil ,G\right). \end{array}$$

Chebyshev's inequality guides the choice of parameter *ℓ*. For *ℓ* ≥ 6, the loss of accuracy due to truncation is negligible.

Computation can be reduced further by recognizing that distributions imposed on the basis of a small number of moment constraints often enjoy a high level of regularity so that a sparser mesh should be adequate. The computation-accuracy trade-off becomes much easier to analyze if the problem is posed as one of learning a density function over a continuous domain, say

(B.4) St={hμ(v,w):  v∈R(F),  w∈R(C), −hμ≤v+w≤1−hμ},

where,  *h*_*μ*_≝*G*/(*μ*_*𝔉*_ + *μ*_*C*_),

(B.5) $$\begin{array}{cc} R\left(F\right) & ≝\text{range}\;\text{of}\;F\\ & =\text{the}\;\text{interval}\;\left[\frac{G}{F_{\min}-\mu_{F}},\frac{G}{F_{\max}-\mu_{F}}\right], \end{array}$$

and similarly for *ℜ*(*C*) (see Figure 6). Note that the continuous domain is scaled by the factor *G* to improve numerical stability. The moment constraints must be scaled accordingly. Let *𝒫*_*c*_ denote the space of feasible distributions *p*(*ξ*_1_, *ξ*_2_). Then, for all *p* ∈ *𝒫*_*c*_:

(B.6) $$\begin{array}{cc} & \overset{}{\underset{S_{t}}{\int }}\xi_{1}^{i}\xi_{2}^{j}p\left(\xi_{1},\xi_{2}\right)\text{d}\xi_{1}\text{d}\xi_{2}=\frac{E\left\{{\left(F-\mu_{F}\right)}^{i}{\left(C-\mu_{C}\right)}^{j}\right\}}{G^{i+j}}≝\eta^{ij}, \end{array}$$

with 0 ≤ *i* + *j* ≤ *J*. In (B.6), (*i*, *j*) = (0,0) corresponds to the constraint ∫_*𝒮*_*t*__ *p*(*ξ*_1_, *ξ*_2_)d*ξ*_1_d*ξ*_2_ = 1, while (*i*, *j*) = (1,0) together with (*i*, *j*) = (0,1) imply that every *p* ∈ *𝒫*_*c*_ has mean [0 0]^*T*^. We are concerned with problems for which *J* ≤ 3. For convenience, we have defined the notation *η* ^*ij*^ as a shorthand for the *scaled *(*i*, *j*) joint central moment of *𝔉* and *C*.

The selection of a unique *p*(*ξ*_1_, *ξ*_2_) is based on the principle of entropy maximization (MAXENT) which seeks a *p* ∈ *𝒫*_*c*_ with maximum information entropy [67]. The information entropy essentially measures the spread of the distribution, and hence, maxent can be seen as a criterion, which, within one's knowledge constraints, maximizes the representation of unknown information (“ignorance")—arguably, a suitable approach for statistical inference.

MAXENT seeks the following solution in *𝒫*_*c*_:

(B.7) $$\begin{array}{c} p^{*}\left(\xi_{1},\xi_{2}\right)=\underset{p\in P_{c}}{\max\;}\left\{-\int_{S_{t}}p\left(\xi_{1},\xi_{2}\right)\ln\left\{p\left(\xi_{1},\xi_{2}\right)\right\}\text{d}\xi_{1}\text{d}\xi_{2}\right\}. \end{array}$$

The solution takes the following exponential form:

(B.8) $$\begin{array}{c} p_{\lambda }\left(\xi_{1},\xi_{2}\right)=\frac{\exp\left\{\sum_{1\leq i+j\leq J}^{}\;\lambda_{ij}\;\;\xi_{1}^{i}\;\;\xi_{2}^{j}\right\}}{\int_{S_{t}}\exp\left\{\sum_{1\leq i+j\leq J}^{}\lambda_{ij}\;\xi_{1}^{i}\;\xi_{2}^{j}\right\}\text{d}\xi_{1}\text{d}\xi_{2}}, \end{array}$$

in which the *λ*_*ij*_ are Lagrange multipliers. The derivation of the exponential form (B.8) and a procedure to determine optimal multipliers *λ*_*ij*_ are given in the following subsection.

#### B.2. MAXENT Solution Details

The information entropy functional is concave [68], and the constraints in (B.6) are linear in *p*(*ξ*_1_, *ξ*_2_). Thus, the problem in (B.7) is a convex program that be solved in a Lagrangian dual framework, where one works with an unconstrained upper bound that is easy to optimize (e.g., [69]). More importantly, in the present case, the framework allows the conversion of the original infinite-dimension problem of functional variation into a finite-dimension problem with as few variables as the number of constraints.

Lemma 4 . The dual, Ψ(***λ***), of the concave optimization problem (B.7) is given by:

(B.9) $$\begin{array}{c} \Psi \left(\lambda \right)=\ln\left[\int_{S_{t}}\exp\left\{\underset{1\leq i+j\leq J}{\sum }\lambda_{ij}\xi_{1}^{i}\xi_{2}^{j}\right\}\text{d}\xi_{1}\text{d}\xi_{2}\right]-\underset{2\leq i+j\leq J}{\sum }\lambda_{ij}\eta^{ij}, \end{array}$$

where ***λ*** is the set of Lagrange multipliers, {*λ*_*ij*_}_*i*,*j*_; *λ*_*ij*_ is the multiplier corresponding to the (*i*, *j*)th constraint; and *i*, *j*, *J*, and *η* ^*ij*^ are defined in (B.6).

Proof By the definition of the Lagrangian dual function

(B.10) Ψ(λ)=sup⁡p∈Pc⁡{∑i+j≤J−∫Stp(ξ1,ξ2)ln⁡{p(ξ1,ξ2)}dξ1dξ2    +∑i+j≤Jλij(∫Stξ1iξ2jp(ξ1,ξ2)dξ1dξ2−ηij)}.

Taking the functional variation of the bracketed term in (B.10) with respect to the unknown density *p*(*ξ*_1_, *ξ*_2_), and using the fact that ∫_*𝒮*_*t*__ *p*(*ξ*_1_, *ξ*_2_) d*ξ*_1_ d*ξ*_2_ = 1, we obtain the maximizer of (B.10)

(B.11) $$\begin{array}{c} p_{\lambda }\left(\xi_{1},\xi_{2}\right)=\frac{\exp\left\{\sum_{1\leq i+j\leq J}^{}\lambda_{ij}\xi_{1}^{i}\xi_{2}^{j}\right\}}{\int_{S_{t}}\exp\left\{\sum_{1\leq i+j\leq J}^{}\lambda_{ij}\xi_{1}^{i}\xi_{2}^{j}\;\right\}\;\text{d}\xi_{1}\;\text{d}\xi_{2}}. \end{array}$$

Inserting (B.11) into (B.10) yields

(B.12) $$\begin{array}{cc} \Psi \left(\lambda \right) & =\int_{S_{t}}p\left(\xi_{1}^{\prime },\xi_{2}^{\prime }\right)\\ & \;\times \ln\left\{\int_{S_{t}}\exp\left(\underset{1\leq i+j\leq J}{\sum }\lambda_{ij}\xi_{1}^{i}\xi_{2}^{j}\right)\text{d}\xi_{1}\text{d}\xi_{2}\right\}\;\text{d}\xi_{1^{\prime }}\text{d}\xi_{2^{\prime }}\\ & \;\;-\underset{1\leq i+j\leq J}{\sum }\lambda_{ij}\eta^{ij}\\ & =\ln\left\{\int_{S_{t}}\exp\left(\underset{1\leq i+j\leq J}{\sum }\lambda_{ij}\xi_{1}^{i}\xi_{2}^{j}\right)\text{d}\xi_{1}\text{d}\xi_{2}\right\}\\ & \;\;-\underset{2\leq i+j\leq J}{\sum }\lambda_{ij}\eta^{ij}, \end{array}$$

where we have used the facts ∫_*𝒮*_*t*__ *p*(*ξ*_1_, *ξ*_2_)d*ξ*_1_ d*ξ*_2_ − *η* ^00^ = 0, *η* ^10^ = 0, and *η* ^01^ = 0 from (B.6).

It is easy to verify that the Hessian, denoted *ℋ*{·}, of (B.9) is positive definite and hence Ψ(***λ***) is convex. Suppose that ***λ*** ^*^ is the minimum of Ψ(***λ***). Then the corresponding *primal solution p*_***λ***^*^_(*ξ*_1_, *ξ*_2_)—obtained via (B.11)—indeed maximizes (B.7). To verify this, let *p*_*o*_(*ξ*_1_, *ξ*_2_) be the maximizer of (B.7). Then, from (B.10), Ψ(***λ***) ≥ *ℋ*{*p*_*o*_(*ξ*_1_, *ξ*_2_)} for all ***λ***. Now from general optimization theory, the functional variation of the Lagrangian with respect to *p*(*ξ*_1_, *ξ*_2_) evaluated at *p*_*o*_(*ξ*_1_, *ξ*_2_) must be zero, which implies that *p*_*o*_(*ξ*_1_, *ξ*_2_) can be written in the form (B.11) for some ***λ***_*o*_. But, then, Ψ(***λ*** ^*^) ≤ Ψ(***λ***_*o*_)⇒Ψ(***λ*** ^*^) ≤ *ℋ*{*p*_*o*_(*ξ*_1_, *ξ*_2_)}. Consequently, Ψ(***λ*** ^*^) = *ℋ*{*p*_*o*_(*ξ*_1_, *ξ*_2_)}, so that ***λ*** ^*^ = ***λ***_*o*_. Hence *p*_***λ***^*^_(*ξ*_1_, *ξ*_2_) is the maximizer of (B.7).

Newton's method (e.g., [70, Section 4.6]) specifies that if a multivariable function Ψ(***λ***) is twice differentiable and the initial value ***λ***_0_ is chosen close enough to the optimal ***λ*** ^*^, then the sequence over indices *t* = 0,1, 2,…,

(B.13) $$\begin{array}{c} \lambda_{t+1}=\lambda_{t}-\gamma \;\frac{\Delta \Psi \left(\lambda_{t}\right)}{H\left\{\Psi \left(\lambda_{t}\right)\right\}} \end{array}$$

converges to ***λ*** ^*^. In (B.13), ΔΨ(***λ***_*t*_) denotes the gradient of Ψ(***λ***) evaluated at ***λ***_*t*_. The parameter *γ* > 0 allows a finer control of step sizes to avoid numerical instabilities. Intuitively, at the *t*th iteration, Ψ(***λ***) is replaced by its second-order Taylor expansion around ***λ***_*t*_ and then minimized exactly, which produces the minimum ***λ***_*t*+1_. At the (*t* + 1)st iteration, ***λ***_*t*+1_ becomes the point of expansion and the method continues until the desired convergence level is achieved.

The elements of the gradient $\Delta \Psi (\overset{˘}{\lambda })$ are given by:

(B.14) $$\begin{array}{cc} & \frac{\partial \Psi \left(\overset{˘}{\lambda }\right)}{\partial \lambda_{ij}}\\ & \;=\int_{S_{t}}\xi_{1}^{i}\xi_{2}^{j}\\ & \;\;\times \left[\frac{\exp\left\{\sum_{1\leq i+j\leq J}^{}{\overset{˘}{\lambda }}_{ij}\xi_{1}^{i}\xi_{2}^{j}\right\}}{\int_{S_{t}}\exp\left\{\sum_{1\leq i+j\leq J}^{}{\overset{˘}{\lambda }}_{ij}\xi_{1}^{i}\xi_{2}^{j}\right\}\;\text{d}\xi_{1}\text{d}\xi_{2}}\right]\times \text{d}\xi_{1}\;\text{d}\xi_{2}-\eta^{ij}\;\\ & \;=\int_{S_{t}}\xi_{1}^{i}\xi_{2}^{j}\overset{˘}{p}\left(\xi_{1},\xi_{2}\right)\text{d}\xi_{1}\text{d}\xi_{2}-\eta^{ij}={\overset{˘}{\eta }}^{ij}-\eta^{ij}, \end{array}$$

where ${\overset{˘}{\eta }}^{ij}$ denotes the (*i*, *j*) central moment of the distribution of (B.11) parameterized by $\overset{˘}{\lambda }$. Similarly, the elements of the Hessian are given by:

(B.15) $$\begin{array}{cc} \frac{\partial^{2}\Psi \left(\overset{˘}{\lambda }\right)}{\partial \lambda_{i^{\prime }j^{\prime }}\partial \lambda_{ij}} & =\int_{S_{t}}\xi_{1}^{i+i^{\prime }}{\xi_{2}}^{j+j^{\prime }}\overset{˘}{p}\left(\xi_{1},\xi_{2}\right)\;\text{d}\xi_{1}\text{d}\xi_{2}\\ & \;-\int_{S_{t}}\xi_{1}^{i^{\prime }}\xi_{2}^{j^{\prime }}\overset{˘}{p}\left(\xi_{1},\xi_{2}\right)\;\text{d}\xi_{1}\text{d}\xi_{2}\\ & \;\times \int_{S_{t}}\xi_{1}^{i}\xi_{2}^{j}\overset{˘}{p}\left(\xi_{1},\xi_{2}\right)\;\text{d}\xi_{1}\text{d}\xi_{2}\\ & ={\overset{˘}{\eta }}^{\left(i+i^{\prime })(j+j^{\prime }\right)}-{\overset{˘}{\eta }}^{i^{\prime }j^{\prime }}{\overset{˘}{\eta }}^{ij}. \end{array}$$

The gradient calculations that occur as a part of the Hessian essentially involve integration over the planar domain *𝒮*_*t*_. Many advanced techniques for implementing numerical integration on a quadrangle like *𝒮*_t_ are available in the literature; however, an equispaced rectangular mesh is found to be sufficient for the present purpose. We initiate the sequence (B.13) with ***λ*** = 0 which implies a uniform distribution over *𝒮*_*t*_.

## References

[1] http://www.genome.gov/.

[2] Page G. P., Zakharkin S. O., Kim K., Mehta T., Chen L., Zhang K. (2007). Microarray analysis. *Methods in Molecular Biology*.

[3] Wang J. (2008). Computational biology of genome expression and regulation—a review of microarray bioinformatics. *Journal of Environmental Pathology, Toxicology and Oncology*.

[4] Efron B. (2007). Size, power and false discovery rates. *Annals of Statistics*.

[5] Efron B. (2004). Large-scale simultaneous hypothesis testing: the choice of a null hypothesis. *Journal of the American Statistical Association*.

[6] Schena M., Shalon D., Davis R. W., Brown P. O. (1995). Quantitative monitoring of gene expression patterns with a complementary DNA microarray. *Science*.

[7] Datta S., Datta S. (2006). Evaluation of clustering algorithms for gene expression data. *BMC Bioinformatics*.

[8] Gibbons F. D., Roth F. P. (2002). Judging the quality of gene expression-based clustering methods using gene annotation. *Genome Research*.

[9] Handl J., Knowles J., Kell D. B. (2005). Computational cluster validation in post-genomic data analysis. *Bioinformatics*.

[10] Sémon M., Duret L. (2006). Evolutionary origin and maintenance of coexpressed gene clusters in mammals. *Molecular Biology and Evolution*.

[11] Li K. C. (2002). Genome-wide coexpression dynamics: theory and application. *Proceedings of the National Academy of Sciences of the United States of America*.

[12] Kluger Y., Basri R., Chang J. T., Gerstein M. (2003). Spectral biclustering of microarray data: coclustering genes and conditions. *Genome Research*.

[13] Tsai C. A., Lee T. C., Ho I. C., Yang U. C., Chen C. H., Chen J. J. (2005). Multi-class clustering and prediction in the analysis of microarray data. *Mathematical Biosciences*.

[14] Tusher V. G., Tibshirani R., Chu G. (2001). Significance analysis of microarrays applied to the ionizing radiation response. *Proceedings of the National Academy of Sciences of the United States of America*.

[15] Choi Y., Kendziorski C. (2009). Statistical methods for gene set co-expression analysis. *Bioinformatics*.

[16] Barry W., Nobel A., Wright F. (2008). A statistical framework for testing functional categories in microarray data. *Annals of Applied Statistics*.

[17] Peters T., Bulger D. W., Loi T.-H., Yang J. Y. H., Ma D. (2011). Two-step cross-entropy feature selection for microarrays-power through complementarity. *IEEE/ACM Transactions on Computational Biology and Bioinformatics*.

[18] Yang F., Mao K. Z. (2011). Robust feature selection for microarray data based on multicriterion fusion. *IEEE/ACM Transactions on Computational Biology and Bioinformatics*.

[19] Tiño G., Zhao H., Yan H. (2011). Searching for coexpressed genes in three-color cDNA microarray data using a probabilistic model-based hough transform. *IEEE/ACM Transactions on Computational Biology and Bioinformatics*.

[20] Ruan J., Dean A. K., Zhang W. (2010). A general co-expression network-based approach to gene expression analysis: comparison and applications. *BMC Systems Biology*.

[21] Dettling M., Gabrielson E., Parmigiani G. (2005). Searching for differentially expressed gene combinations. *Genome Biology*.

[22] Lai Y., Wu B., Chen L., Zhao H. (2004). A statistical method for identifying differential gene-gene co-expression patterns. *Bioinformatics*.

[23] Ng Y. K., Wu W., Zhang L. (2009). Positive correlation between gene coexpression and positional clustering in the zebrafish genome. *BMC Genomics*.

[24] Bernthaler I., Mühlberger A., Fechete R., Perco P., Lukas A., Mayer B. Interpreting microarray experiments via Co-expressed Gene Groups Analysis (CGGA).

[25] Reverter A., Chan E. K. F. (2008). Combining partial correlation and an information theory approach to the reversed engineering of gene co-expression networks. *Bioinformatics*.

[26] Tchagang A. B., Tewfik A. H. (2006). DNA microarray data analysis: a novel biclustering algorithm approach. *Eurasip Journal on Applied Signal Processing*.

[27] Martinez R., Pasquier N., Pasquier C., Lopez-Perez L. (2009). A dependency graph approach for the analysis of differential gene expression profiles. *Molecular BioSystems*.

[28] Tsai C. A., Chen J. J. (2009). Multivariate analysis of variance test for gene set analysis. *Bioinformatics*.

[29] Pan W. (2002). A comparative review of statistical methods for discovering differentially expressed genes in replicated microarray experiments. *Bioinformatics*.

[30] Owen A. B. (2005). Variance of the number of false discoveries. *Journal of the Royal Statistical Society. Series B*.

[31] Efron B. (2007). Correlation and large-scale simultaneous significance testing. *Journal of the American Statistical Association*.

[32] Pawitan Y., Murthy K. R. K., Michiels S., Ploner A. (2005). Bias in the estimation of false discovery rate in microarray studies. *Bioinformatics*.

[33] Leek J. T., Storey J. D. (2007). Capturing heterogeneity in gene expression studies by surrogate variable analysis. *PLoS Genetics*.

[34] Degrelle S. A., Hennequet-Antier C., Chiapello H. (2008). Amplification biases: possible differences among deviating gene expressions. *BMC Genomics*.

[35] Qiu X., Klebanov L., Yakovlev A. (2005). Correlation between gene expression levels and limitations of the empirical Bayes methodology for finding differentially expressed genes. *Statistical Applications in Genetics and Molecular Biology*.

[36] Qiu X., Yakovlev A. (2006). Some comments of instability of false discovery rate estimation. *Journal of Bioinformatics and Computational Biology*.

[37] Storey J. D., Dai J. Y., Leek J. T. (2007). The optimal discovery procedure for large-scale significance testing, with applications to comparative microarray experiments. *Biostatistics*.

[38] Tibshirani R., Wasserman L. Correlation-sharing for detection of differential gene expression. http://arxiv.org/abs/math/0608061.

[39] Hu R., Qiu X., Glazko G. (2010). A new gene selection procedure based on the covariance distance. *Bioinformatics*.

[40] Cui Q., Liu B., Jiang T., Ma S. (2005). Characterizing the dynamic connectivity between genes by variable parameter regression and Kalman filtering based on temporal gene expression data. *Bioinformatics*.

[41] Martyanov V., Gross R. H. (2010). Identifying functional relationships within sets of co-expressed genes by combining upstream regulatory motif analysis and gene expression information. *BMC Genomics*.

[42] Tewhey R., Bansal V., Torkamani A., Topol E. J., Schork N. J. (2011). The importance of phase information for human genomics. *Nature Reviews Genetics*.

[43] Xiang Z., Qin Z. S., He Y. (2007). CRCView: a web server for analyzing and visualizing microarray gene expression data using model-based clustering. *Bioinformatics*.

[44] Wong A. K. C., Au W. H., Chan K. C. C. (2008). Discovering high-order patterns of gene expression levels. *Journal of Computational Biology*.

[45] Bandyopadhyay S., Bhattacharyya M. (2011). A biologically inspired measure for co-expression analysis. *IEEE Transactions Computational Biology and Bioinformatics*.

[46] Dalton L., Ballarin V., Brun M. (2009). Clustering algorithms: on learning, validation, performance, and applications to genomics. *Current Genomics*.

[47] Ancona N., Maglietta R., Piepoli A. (2006). On the statistical assessment of classifiers using DNA microarray data. *BMC Bioinformatics*.

[48] Hu T., Peng H., Sun W. (2011). Incorporating nonlinear relationships in microarray missing value imputation. *IEEE/ACM Transactions on Computational Biology and Bioinformatics*.

[49] Hsueh H. M., Tsai C. A., Chen J. J. (2007). Incorporating the number of true null hypotheses to improve power in multiple testing: application to gene microarray data. *Journal of Statistical Computation and Simulation*.

[50] Langaas M., Lindqvist B. H., Ferkingstad E. (2005). Estimating the proportion of true null hypotheses, with application to DNA microarray data. *Journal of the Royal Statistical Society. Series B*.

[51] Storey J. D., Taylor J. E., Siegmund D. (2004). Strong control, conservative point estimation and simultaneous conservative consistency of false discovery rates: a unified approach. *Journal of the Royal Statistical Society. Series B*.

[52] Waterman M., Whiteman D. (1978). Estimation of probability densities by empirical density functions. *Journal of Mathematical Education in Science and Technology*.

[53] Bolstad B. M., Irizarry R. A., Åstrand M., Speed T. P. (2003). A comparison of normalization methods for high density oligonucleotide array data based on variance and bias. *Bioinformatics*.

[54] Qiu X., Brooks A. I., Klebanov L., Yakovlev A. (2005). The effects of normalization on the correlation structure of microarray data. *BMC Bioinformatics*.

[55] Hotelling H. (1953). New light on the correlation coefficient and its transforms. *Journal of the Royal Statistical Society*.

[56] Wishart J. (1928). The generalised product moment distribution in samples from a normal multivariate population. *Biometrika*.

[57] Barnard J., McCulloch R., Meng X. L. (2000). Modeling covariance matrices in terms of standard deviations and correlations, with application to shrinkage. *Statistica Sinica*.

[58] Olkin I. (1953). Note on ‘The Jacobians of certain matrix transformations useful in multivariate analysis’. *Biometrika*.

[59] Liechty J., Liechty M., Muller P. (2004). Bayesian correlation estimation. *Biometrika*.

[60] Hedenfalk I., Duggan D., Chen Y. (2001). Gene-expression profiles in hereditary breast cancer. *New England Journal of Medicine*.

[61] Tsai C. A., Chen Y. J., Chen J. J. (2003). Testing for differentially expressed genes with microarray data. *Nucleic Acids Research*.

[62] Van 't Wout A. B., Lehrman G. K., Mikheeva S. A. (2003). Cellular gene expression upon human immunodeficiency virus type 1 infection of CD4+-T-cell lines. *Journal of Virology*.

[63] Lehmann E. L., Romano J. P. (2005). Generalizations of the familywise error rate. *Annals of Statistics*.

[64] Newton M. A., Kendziorski C. M., Richmond C. S., Blattner F. R., Tsui K. W. (2001). On differential variability of expression ratios: improving statistical inference about gene expression changes from microarray data. *Journal of Computational Biology*.

[65] Golub G., Van Loan C. (1996). *Matrix Computations*.

[66] Qi H., Sun D. (2006). A quadratically convergent Newton method for computing the nearest correlation matrix. *SIAM Journal on Matrix Analysis and Applications*.

[67] Jaynes E. (2003). *Probability Theory: The Logic of Science*.

[68] Cover T., Thomas J. (1991). *Elements of Information Theory*.

[69] Gelfand I., Fomin S. (2000). *Calculus of Variations [English translation]*.

[70] Haykin S. (2002). *Adaptive Filter Theory*.

